# Strong nonlinear optical processes with extraordinary polarization anisotropy in inversion-symmetry broken two-dimensional PdPSe

**DOI:** 10.1038/s41377-024-01474-6

**Published:** 2024-05-27

**Authors:** Song Zhu, Ruihuan Duan, Xiaodong Xu, Fangyuan Sun, Wenduo Chen, Fakun Wang, Siyuan Li, Ming Ye, Xin Zhou, Jinluo Cheng, Yao Wu, Houkun Liang, Junichiro Kono, Xingji Li, Zheng Liu, Qi Jie Wang

**Affiliations:** 1 School of Electrical and Electronic Engineering, Nanyang Technological University, 639798 Singapore, Singapore; 2 School of Material Science and Engineering, Nanyang Technological University, 639798 Singapore, Singapore; 3 CINTRA CNRS/NTU/THALES, UMI 3288, Research Techno Plaza, Nanyang Technological University, 637371 Singapore, Singapore; 4 School of Materials Science and Engineering, Harbin Institute of Technology, 150001 Harbin, China; 5 Department of Chemistry, National University of Singapore, 117543 Singapore, Singapore; 6 GPL Photonics Lab, State Key Laboratory of Applied Optics, Changchun Institute of Optics, Fine Mechanics and Physics, Chinese Academy of Sciences, 130033 Changchun, China; 7 School of Electronics and Information Engineering, Sichuan University, 610064 Chengdu, Sichuan China; 8 School of Physical and Mathematical Sciences, Nanyang Technological University, 637371 Singapore, Singapore; 9 Departments of Electrical and Computer Engineering, Physics and Astronomy, and Materials Science and NanoEngineering, Rice University, Houston, TX USA

**Keywords:** Optical properties and devices, Carbon nanotubes and fullerenes

## Abstract

Nonlinear optical activities, especially second harmonic generation (SHG), are key phenomena in inversion-symmetry-broken two-dimensional (2D) transition metal dichalcogenides (TMDCs). On the other hand, anisotropic nonlinear optical processes are important for unique applications in nano-nonlinear photonic devices with polarization functions, having become one of focused research topics in the field of nonlinear photonics. However, the strong nonlinearity and strong optical anisotropy do not exist simultaneously in common 2D materials. Here, we demonstrate strong second-order and third-order susceptibilities of 64 pm/V and 6.2×10^−19^ m^2^/V^2^, respectively, in the even-layer PdPSe, which has not been discovered in other common TMDCs (e.g., MoS_2_). Strikingly, it also simultaneously exhibited strong SHG anisotropy with an anisotropic ratio of ~45, which is the largest reported among all 2D materials to date, to the best of our knowledge. In addition, the SHG anisotropy ratio can be harnessed from 0.12 to 45 (375 times) by varying the excitation wavelength due to the dispersion of ${\chi }^{(2)}$ values. As an illustrative example, we further demonstrate polarized SHG imaging for potential applications in crystal orientation identification and polarization-dependent spatial encoding. These findings in 2D PdPSe are promising for nonlinear nanophotonic and optoelectronic applications.

## Introduction

Nonlinear optics has been a fundamental building block of modern optics and lies at the core of laser technology, optical spectroscopy, and quantum networks^1,2^. Atomically thin van der Waals two-dimensional (2D) crystals such as graphene and TMDCs provide an excellent platform for nonlinear optical responses in two dimensions^3–14^ owing to their extraordinary nonlinear optical properties in contrast to bulk materials^15^. Second-harmonic generation (SHG) and third-harmonic generation (THG) processes in 2D materials are emerging branches of nonlinear optics and have been widely exploited for various applications, such as nonlinear optical modulators^16–18^, nonreciprocal optical devices^19,20^, and nonlinear optical imaging^21–23^. In contrast to THG, the SHG process can only be generated in systems with broken inversion symmetry^24–26^. Up to now, several methods have been proposed to generate SHG in 2D materials by breaking the inversion symmetry. Odd-layer TMDCs such as molybdenum disulfide (MoS_2_) and molybdenum ditelluride (MoTe_2_) were found to possess broken inversion symmetry and display strong SHG^27,28^. Furthermore, few expanded approaches have been proposed to generate SHG by breaking the inversion symmetry via gate engineering^17,29^, twist-angle and layer-stacking engineering^30,31^, and interlayer excitons^32^.

Optical anisotropy is envisioned as a new degree of freedom to manipulate and modulate light beams leveraging on birefringence induced by asymmetric crystal structures^33–40^. Anisotropic nonlinear optical responses generated from the difference of polarization ***P***(*t*) in two crystal orientations have become a recent research focus, such as anisotropic nonlinear absorption^41^, anisotropic SHG^42^, and anisotropic THG^43,44^ in asymmetric 2D materials. Such anisotropic SHG and THG processes can find unique applications in nano-nonlinear photonic devices, such as optical switching^45^, polarized pulsed lasers^46^, bio-microscopy^47^, phase-match elements^48,49^, and crystal orientation identification^38^. Li et al. reported a linearly polarized pulsed laser with a high polarization degree of 98% using 2D black phosphorus with the high anisotropic third-order nonlinear effect^46^. However, unfortunately, strong nonlinear optical anisotropy and strong nonlinearity do not appear simultaneously in current common 2D materials such as gallium selenide (GaSe)^50^, MoS_2_^51^_,_ and rhenium disulfide (ReS_2_)^42^. Therefore, exploring new inversion-symmetry-broken 2D materials with the coexistence of strong nonlinear optical anisotropy and strong optical nonlinearity (including SHG and THG) is highly desirable for fundamental research as well as novel optoelectronic applications. Recently, a new class of 2D pentagonal material, penta-PdPSe with low-symmetry lattice characteristics has attracted increasing research attention^52^. However, there has been no research in nonlinear optical properties of 2D PdPSe materials up to now.

Here, we describe our finding that even-layer PdPSe has strong inversion-symmetry-broken behaviors, which are distinct from other common TMDCs^7,51^, with a strong second-order susceptibility (${\chi }^{(2)}$) of 64 pm/V and a third-order susceptibility (${\chi }^{(3)}$) of 6.2 × 10^−19^ m^2^/V^2^. These PdPSe flakes also exhibit a strong SHG anisotropic ratio of ~45 with the *b*-axis being the high SHG polarization orientation. We show that the measured SHG and THG processes exhibit strong and unique wavelength- and layer-dependent anisotropic ratios in 2D PdPSe. In particular, the SHG anisotropy ratio can be harnessed by 375 times by choosing the excitation wavelength. We also performed polarized SHG imaging as a demonstration example, showing great potentials for crystal orientation identification and polarization-dependent spatial encoding. The coexistence of strong nonlinear optical anisotropy and strong nonlinear optical response in 2D PdPSe is promising for a wide variety of nonlinear photonic and optoelectronic applications.

## Results

### Crystal structure and characterization of the PdPSe crystal

**Fig. 1:**
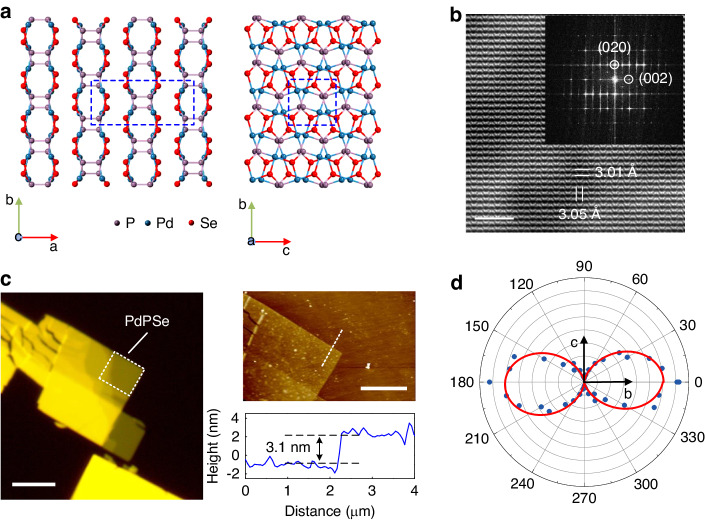
**Anisotropic electron and phonon characterization of the 2D PdPSe crystal**. **a** The lattice structure of 2D layered PdPSe with a puckered pentagonal. Left: side view of 4 L PdPSe; Right: top view of the atomic crystal structure of monolayer PdPSe. **b** HRTEM image of the few-layer PdPSe. Inset: the FFT pattern of Fig. 1b. Scale bar: 2 nm. **c** Left: The optical microscopic image of a few-layer PdPSe on the fused silica substrate. Scale bar: 5 μm; Right upper: AFM image. Scale bar: 5 μm; Right bottom: a cross-sectional profile of the edge of the PdPSe flake marked by white dashed line in the AFM image. **d** Polar plot of the Raman intensities for the${A}_{g}^{4}$ peak as a function of the polarization angle with the parallel configuration (Fig. S3b)

The PdPSe crystal possesses a novel puckered 2D structure, which belongs to orthorhombic space group Pbcn (No. 60): *a* = 13.594 Å, *b* = 5.832 Å, and *c* = 5.858 Å^52^. It has a stratiform structure identical to other van der Waals 2D materials (the left part of Fig. 1a). In contrast to puckered PdSe_2_, whose monolayer is a single-atom puckered pentagonal layer^53^, monolayer PdPSe has a unique double-atom puckered pentagonal structure, in which two sublayers construct a monolayer via the P-P bonds. We performed high-resolution transmission electron microscopy (HRTEM) characterization, which clearly revealed the atomic structure of the PdPSe crystal. The periodic atomic arrangement in the PdPSe crystal can be observed, in which two in-plane lattice constants are measured to be *b* = 5.8317 Å and *c* = 5.8583 Å (Fig. 1b). Two perpendicular lattices show different atomic arrangements, which reveals its strong in-plane anisotropy in the crystal structure. The fast Fourier transform (FFT) diffraction patterns (the inset of Fig. 1b) show clear diffraction spots corresponding to the two principal crystal planes of (002) and (020). We also performed X-ray diffraction (XRD) and energy dispersive analytical X-ray spectroscopy (EDX) measurements to verify the high quality of the crystal (Notes S1 and S2). For the PdPSe crystal, it is difficult to exfoliate monolayer or few layers from the bulk crystal using the conventional scotch tape technique. We obtained large-size few-layer PdPSe flakes using the gold-mediated exfoliation method (“Methods” and Fig. S3a)^54^. Figure 1c shows a typical optical microscopic image and corresponding atomic force microscope (AFM) measurements of a typical PdPSe flake. The flake has a thickness of ~3.1 nm (4 L). To check its anisotropic phonon properties, we performed polarization-dependent Raman spectroscopy measurements with the parallel configuration. The Raman peak (${A}_{{\rm{g}}}^{4}$) intensity shows a periodic variation with a period of 180°, while the maximum intensity is along the *b*-axis of the PdPSe crystal and the minimum intensity is along the *c*-axis, clearly showing its anisotropy (Figs. 1d and S3b).

### Strong SHG and THG responses in 2D PdPSe

**Fig. 2:**
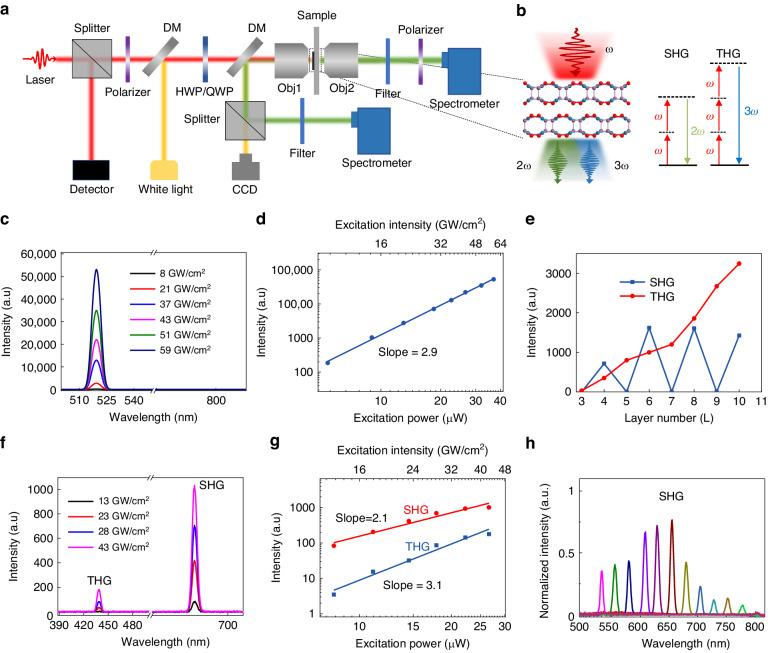
**SHG and THG processes in 2D PdPSe**. **a** Schematic diagram of the THG and SHG experimental setup. **b** Left: Schematic illustration of the SHG and THG processes in the inversion symmetry broken 2D PdPSe; Right: the schematic diagram for the SHG and THG processes. **c** THG spectra depending on the excitation intensity for the PdPSe flake with a thickness of 28 nm under 1550-nm excitation. **d** Excitation power (intensity) dependent THG intensity in the bulk PdPSe with a thickness of 28 nm. **e** SHG and THG intensities as functions of the layer number (L). **f** SHG and THG spectra of 6 L PdPSe under 1300-nm excitation. The peaks at 433 and 650 nm correspond to the THG and SHG signals, respectively. **g** Excitation power (intensity) dependent SHG and THG intensities in 6 L PdPSe. **h** The SHG spectra with the excitation wavelength from 1064 to 1600 nm in 4 L PdPSe

We performed nonlinear optical measurements with a home-built setup (Fig. 2a). Bulk PdPSe crystal belongs to the *D*_*2h*_ (*mmm*) point group, which possesses an inversion symmetry and lacks SHG response. Figure 2c depicts a set of THG spectra measured at various excitation intensity under ~1550-nm excitation. The sample used for these measurements has a thickness of 28 nm (Fig. S6). It can be found that only the THG signal centered at 516 nm can be observed as the excitation intensity increases. We cannot see SHG since the bulk PdPSe has the inversion symmetry. Furthermore, a natural logarithm plot of the THG intensity as a function of the excitation power displays an exponent of ~2.9, indicating the THG process (Fig. 2d)^55^. We theoretically found that the even-layer PdPSe crystal possesses a *C*_2*v*_ (*mm*2) point group in *P*ca21space group (No. 29) with broken inversion symmetry, while the odd-numbered layer belongs to the *C*_2*h*_ (2/*m*) point group in the *P*21/c space group (No.14) with the inversion symmetry (Table S1 and Fig. S8), which is similar to what was observed in layered PdSe_2_^53^. Since the inversion symmetry is layer-number-dependent for few-layer PdPSe, the SHG process should be strongly dependent on the layer number. Figure 2e shows that the SHG signal exhibits the strong intensity in the even layers (4 L, 6 L, 8 L, 10 L), while vanishes in the odd layers (3 L, 5 L, 7 L, 9 L), which means that there is neglected surface SHG in few-layer PdPSe. We also did calculations to prove there is no obvious surface SHG in PdPSe (Figs. S17 and S18). The phenomenon shows a unique layer dependence of SHG in 2D PdPSe, which is different from the TMDCs, in which only odd layers possess the SHG process^6,7,51^. We used a spatial imaging technique to scan the SHG signal in a large-size PdPSe flake, which clearly shows that SHG can only be detected from even-layer PdPSe flakes (Fig. S5). In contrast, THG always exists regardless of the layer number. We find that the THG intensity always increases with the increment of the layer number (Fig. 2e). The SHG intensity decreases a little when the layer number is larger than 6 L, which is attributed to absorption of the SHG signal by the few-layer PdPSe flakes^27,53^. Figure 2f shows the evolution of the SHG and THG spectra with the increment of the excitation intensity under 1300-nm excitation for a 6 L PdPSe. Figure 2g shows log–log plots of the SHG (433 nm) and THG (650 nm) intensities as a function of the excitation power. As expected, the SHG and THG intensities show quadratic and cubic dependence on the excitation power, respectively.

The excitation wavelength is a significant factor influencing the SHG intensity in 2D materials. Figure 2h presents the SHG spectra of 4 L PdPSe with different excitation wavelengths under the same average excitation power of ~10 μW (16 GW/cm^2^). Strikingly, the SHG intensity is strongly dependent on the excitation wavelength from 1064 to 1600 nm and shows the maximum intensity at ~1300 nm, indicating the resonance-enhancement effect induced by the interband transition^15,55–57^, which agrees well with the absorption spectra and calculated band structures of few-layer PdPSe (Figs. S9 and S10). Furthermore, the ${\chi }^{(2)}$ value (${\chi }_{yxx}^{(2)}$) for 6 L PdPSe at 1300 nm is estimated to be 64 pm/V (Note S11). The value is stronger than those of many other 2D materials and many commercial bulk crystals (Table S2). In addition, the ${\chi }^{(3)}$ value of PdPSe is estimated to be 6.2 × 10^−19^ m^2^/V^2^ at ~1550 nm, which is as strong as those in many other 2D materials and many bulk materials (Table S4). The air-stable 2D PdPSe makes it promising for integrating with on-chip photonic devices for nonlinear frequency conversion.

### Strong nonlinear optical anisotropy in 2D PdPSe

Importantly, anisotropic nonlinear optical responses for SHG and THG processes were also studied. Even-layer PdPSe with C_*2v*_ (*mm*2) point group has seven independent SHG susceptibility tensor elements: ${\chi }_{yxx}^{(2)}$, ${\chi }_{yyy}^{(2)}$, ${\chi }_{yzz}^{(2)}$, ${\chi }_{xyx}^{(2)}={\chi }_{xxy}^{(2)}$ and ${\chi }_{zzy}^{(2)}={\chi }_{zyz}^{(2)}$^1^. The parallel (${I}_{\parallel }$) and perpendicular (${I}_{\perp }$) components of the SHG intensity could be described as (Note S12):

1 $${I}_{\parallel }\propto {(2{\chi }_{{xxy}}^{\left(2\right)}{cos} ^{2}\left(\theta \right){sin} \left(\theta \right)+{\chi }_{{yxx}}^{\left(2\right)}{cos }^{2}\left(\theta \right){sin} \left(\theta \right)+{\chi }_{{yyy}}^{\left(2\right)}{sin }^{3}\left(\theta \right))}^{2}$$

2 $${I}_{\perp }\propto {(2{\chi }_{{xxy}}^{\left(2\right)}{cos} \left(\theta \right){sin }^{2}\left(\theta \right)-{\chi }_{{yxx}}^{\left(2\right)}{cos }^{3}\left(\theta \right)-{\chi }_{{yyy}}^{\left(2\right)}{cos (\theta )sin }^{2}\left(\theta \right))}^{2}$$

**Fig. 3:**
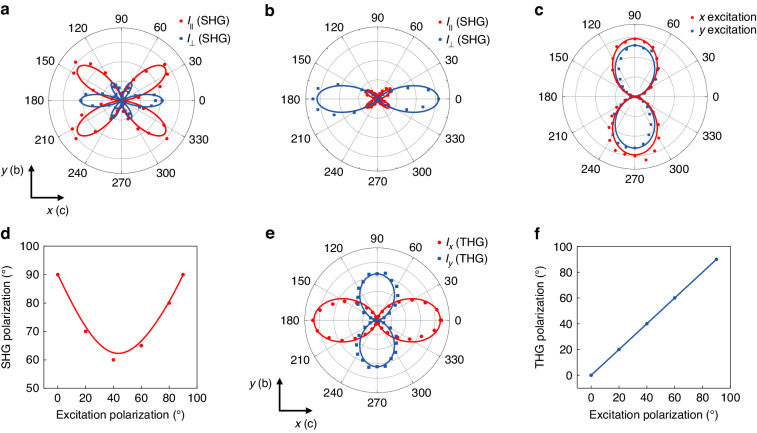
**Anisotropic SHG and THG processes in 6** **L 2D PdPSe**. **a**, **b** Polar plots of the SHG intensity versus the polarization angle (*θ*) with parallel (red) and perpendicular (blue) configurations under 1550-nm and 980-nm excitations. **c** SHG polarization orientation with excitation polarization along *x*-(red) and *y*-(blue) axes of PdPSe under 1300-nm excitation. **d** SHG polarization orientation depending on the excitation polarization. **e** Polar plots of angular dependence of the THG intensity on the excitation polarization at 1550 nm. 0° corresponds to the *x*-axis. Red (blue) data points are measured *x* (*y*)-component of the THG signal, respectively. The solid curves are corresponding theoretical fittings. **f** THG polarization orientation depending on the excitation polarization

Figure 3a shows the polar plots for the angle (*θ*)-dependent SHG of 6 L PdPSe under 1550-nm excitation with the parallel and perpendicular configurations. The maximum value is located on the perpendicular component at angle *θ* = 0, corresponding to the element ${\chi }_{yxx}^{(2)}$. We extracted relative magnitudes of the SHG tensor components as ${\chi }_{xxy}^{(2)}$ :${\chi }_{yxx}^{(2)}$:${\chi }_{yyy}^{(2)}$ = 1:1.5:0.7 by fitting the experimental results using Eqs. 1 and 2, which proved the anisotropic SHG response of 2D PdPSe. Figure 3b shows polar plots for the angle-dependent SHG under a 980-nm excitation. The extracted relative magnitudes of the SHG tensor components are ${\chi }_{xxy}^{(2)}$: ${\chi }_{yxx}^{(2)}$:${\chi }_{yyy}^{(2)}$ = 0.15:1.5:0.3, which is different from that under the 1550-nm excitation, revealing its wavelength-dependent SHG anisotropy. The difference between Fig. 3a, b is derived from the dispersion of ${\chi }^{(2)}$ values, which is due to two-photon absorption at 980 nm along the *c*-axis (Figs. S13 and S19). It is worth noting that the SHG polarization is always along the *y*-axis (*b*-axis) no matter the excitation polarization is along *x*-axis (*c*-axis) or *y*-axis (*b*-axis) (Fig. 3c). Furthermore, the SHG polarization decreases from 90° to 60° and then increases to 90° when the excitation polarization is rotated from 0 to 90° (Fig. 3d). This feature is determined by the unique SHG tensor of even-layer PdPSe (Note S12). Anisotropic THG in few-layer PdPSe was also investigated as shown in Fig. 3e. Red data points depict the *x*-polarized component (*I*_*x*_) of the THG intensity in 6 L PdPSe as a function of the excitation polarization angle relative to the *x*-axis (*c*-axis) under the 1550-nm excitation, while the blue data points correspond to the *y*-polarized component (*I*_*y*_). Red and blue solid lines are the fittings using the extracted relative magnitudes of ${\chi }^{(3)}$ tensor elements ${\chi }_{11}$:${\chi }_{22}$:${\chi }_{18}$:${\chi }_{29}$ = 1:0.85:0.27:0.3 through Eqs. S14 and S15, which shows the anisotropic THG process. Furthermore, THG polarization orientations are always in consistent with the excitation polarization along the *x*- and the *y-*axes (Fig. 3f), which is different from the results of the SHG process (Fig. 3d).

**Fig. 4:**
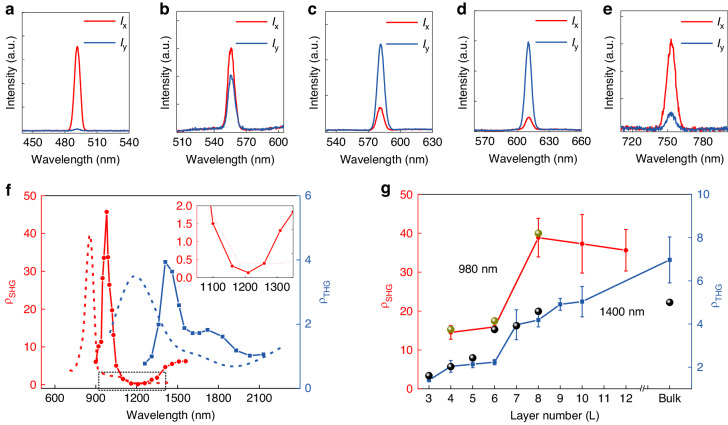
**Anisotropic ratios versus the excitation wavelength and the layer number**. **a**–**e** SHG spectra (8 L PdPSe) for the excitation polarization along *x* (*y*)-axis (*I*_*x*_ and *I*_*y*_) with different excitation wavelengths (**a** 980 nm; **b** 1100 nm; **c** 1150 nm; **d** 1210 nm; **e** 1510 nm). **f** ${\rho }_{SHG}$ (red) and ${\rho }_{THG}$ (blue) versus the excitation wavelength for 8 L PdPSe. Red (blue) dashed lines represent calculated ${\rho }_{SHG}$(${\rho }_{THG}$) depending on the excitation wavelength. Inset: the amplified spectral region marked by the dashed rectangle. **g** ${\rho }_{SHG}$ (red) and ${\rho }_{THG}$ (blue) as functions of the layer number under 980- and 1400-nm excitations, respectively. Green (at 850 nm) and black (at 1190 nm) dots represent calculated results

The anisotropic ratio is a key parameter in the anisotropic nonlinear optical response. The anisotropic ratio is defined as $\rho =\frac{{I}_{x}}{{I}_{y}}$, where *I*_*x*_ is the SHG (THG) intensity with the excitation polarization along the *x*-axis, *I*_*y*_ is the SHG (THG) intensity with the excitation polarization along the *y*-axis. SHG anisotropic ratio (${\rho }_{SHG}$) is determined by the square of the ratio between ${\chi }_{yxx}^{(2)}$ and ${\chi }_{y{\rm{yy}}}^{(2)}$. In order to study influence of ${\chi }^{(2)}$ and ${\chi }^{(3)}$ dispersions on the nonlinear anisotropy, it is desirable to study wavelength dependent ${\rho }_{SHG}$ and ${\rho }_{THG}$ values. Figure 4a–e display SHG spectra with *x*- and *y*-excitations at different wavelengths. It is found that the ${\rho }_{SHG}$ value strongly depends on the excitation wavelength from 980 to 1510 nm and has high values around 980 nm with the maximum value of ~45, corresponding to two elements ratio $\frac{{\chi }_{yxx}^{(2)}}{{\chi }_{yyy}^{(2)}}=6.7:1$ (Fig. 4a). The strong SHG anisotropy under 980 nm-excitation is due to two-photon resonance (absorption peak at 490 nm in Fig. S9) around Y-point in the Brillouin zone (For detailed analysis, see Fig. S19). To the best of our knowledge, the ${\rho }_{SHG}$ value demonstrated in this work is the largest in the atomic layered 2D materials, which is out of resonance at this spectral region (Table S3). Importantly, we find that ${\rho }_{SHG} > 1$ when the wavelength is smaller than ~1100 nm and larger than ~1300 nm, and ${\rho }_{SHG} < 1$ when the wavelength is in between ~1100 and ~1300 nm (Fig. 4c, d). The wavelength dependent ${\rho }_{SHG}$ makes it possible to harness ${\rho }_{SHG}$ over a large value range by varying the excitation wavelength (Fig. 4f) (For detailed analysis, see Fig. S19.). Furthermore, 8 L PdPSe shows a high ${\rho }_{SHG}$ value of 0.12 under ~1200-nm excitation, corresponding to $\frac{{\chi }_{yxx}^{(2)}}{{\chi }_{yyy}^{(2)}}=1:0.35$. In a word, ${\rho }_{SHG}$ value can be harnessed by up to 375 times by choosing the excitation wavelength. ${\rho }_{SHG}$ values for different even layers were also studied. ${\rho }_{SHG}$ values in 4 L and 6 L show high ${\rho }_{SHG}$ values between 10 and 20 and increase to the highest values for the thicker even layers (Fig. 4g). For the thicker layers larger than 8 L, ${\rho }_{SHG}$ always maintains between 30 and 40, which is because the band gap of PdPSe changes little when the layer number is large enough (Fig. S10h) (For detailed analysis, see Fig. S20). The THG anisotropic ratio $\left({\rho }_{THG}=\frac{{I}_{x}}{{I}_{y}}\propto {\left(\frac{{\chi }_{11}^{(3)}}{{\chi }_{22}^{(3)}}\right)}^{2}\right)$ of 2D PdPSe was also studied as presented in Fig. 4f. The blue curve shows that the${\rho }_{THG}$ value strongly depends on the excitation wavelength and displays the maximum value of ~4 under ~1400-nm excitation, corresponding to $\frac{{\chi }_{11}^{(3)}}{{\chi }_{22}^{(3)}}=2:1$. The strong THG anisotropy under 1400-nm excitation is mainly contributed by the three-photon nonlinear process taking places around S-point in the Brillouin zone, which is marked with the blue arrow in the band structure (For detailed analysis, see Fig. S22.). The blue curve shows the ${\rho }_{THG}$ value depending on the layer number (Fig. 4g). It is noted that the ${\rho }_{THG}$ value increases with the layer number and shows the maximum value of ~7, corresponding to $\frac{{\chi }_{11}^{(3)}}{{\chi }_{22}^{(3)}}=2.6:1$, which is the largest demonstrated so far in the layered 2D materials (Table S5). The monotonic increase in ${\rho }_{THG}$ with the layer number is due to a fact that the transition dipole moment (TDM) for the three-photon absorption increases with the layer number (For detailed analysis, see Fig. S22.). The theoretical ${\rho }_{SHG}$ (red dashed line) and ${\rho }_{THG}$ (blue dashed line) as a function of the excitation wavelength agree with the measured results in the intensity and the trend. The difference in peak position between the experimental results and the calculations likely attributes to the Hubbard on-site energy U = 7 eV, which is used to benchmark with the experimental bandgap of the bulk, inevitably inducing a small difference for the estimation of bandgaps of layered slabs. The nonzero components of SHG susceptibilities of 2 L, 4 L, 6 L and 8 L are shown in Fig. S13. A cross point between ${\chi }_{yxx}^{(2)}$ and ${\chi }_{yyy}^{(2)}$ is observed around 1.1 eV (Fig. S13d), which can interpret the inversion behavior of ${\rho }_{SHG}$. By comparison, components ${\chi }_{yzz}^{(2)}$ and ${\chi }_{zzy}^{(2)}$=${\chi }_{zyz}^{(2)}$ are much smaller than ${\chi }_{xyx}^{(2)}$=${\chi }_{x{\rm{xy}}}^{(2)}$, ${\chi }_{yxx}^{(2)}$ and ${\chi }_{{\rm{yyy}}}^{(2)}$. The components ${\chi }_{11}^{(3)}$ and ${\chi }_{22}^{(3)}$ of THG susceptibilities are shown in Fig. S14. Within the photon energy ranging from 0.8 eV to 2 eV, the value of ${\chi }_{11}^{(3)}$ is larger than that of ${\chi }_{22}^{(3)}$, verifying the THG anisotropy observed in the experiment. There are agreements between the experimental and calculated results for layer-dependent anisotropic ratios for both SHG and THG (see the green (at 850 nm) and black (at 1190 nm) dots for the calculated results in Fig. 4g).

The remarkable nonlinear optical responses of PdPSe layers were interpreted by using an efficient nonlinear optical simulation method, which is based on the density functional theory (DFT) in combination with the Wannier interpolation. To obtain an accurate band dispersion in the first Brillouin zone, we firstly conducted DFT calculations of the SCAN + U method for determining the ground states of PdPSe layers. Using the Wannier inperpolation method, a tight-binding Hamiltonian was built to completely reproduce the band dispersions of DFT results. The band structures of 1 L–8 L and bulk PdPSe are shown in Fig. S10a–g, of which the indirect band gaps decrease from 1.86 to 1.42 eV with the increase of the thickness in line with the experimental results (Fig. S10h)^52^. The nonlinear optical simulations are fully based on the constructed Hamiltonian.

### Polarized SHG spatial imaging

**Fig. 5:**
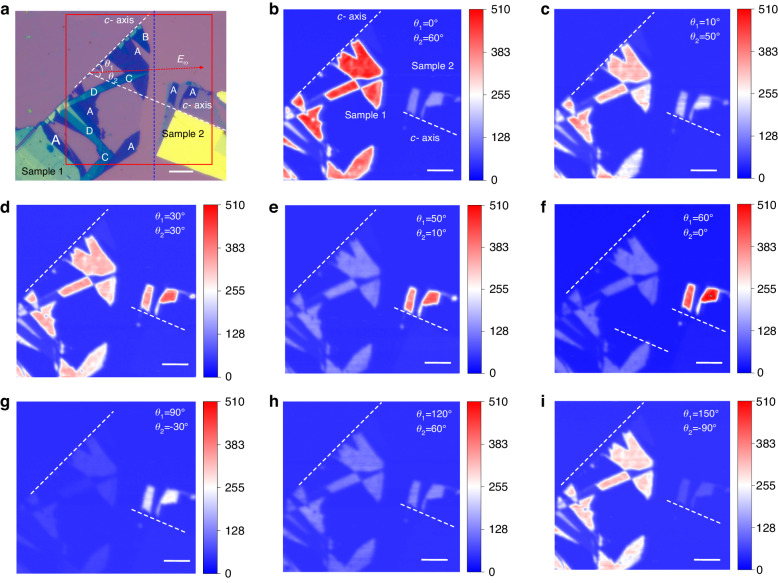
**Polarized SHG spatial imaging from anisotropic few-layer PdPSe**. **a** Optical microscopic images of the PdPSe flakes; A: 6 L, B: 7 L, C: 8 L, D: 9 L; Sample 1 and Sample 2 are in the left and right of the blue dashed line, respectively; Red dashed arrow represents the excitation polarization orientation, which has angles of *θ*_1_ and *θ*_2_ with respect to Sample 1 and Sample 2, respectively; Spatial imaging of the SHG intensity is marked by the red rectangle. Scale bar: 10 μm. **b**–**i** SHG spatial imaging of the PdPSe flakes excited by the 980-nm laser with different polarizations. Scale bar: 10 μm

As mentioned in the previous part, even-layer 2D PdPSe possess a high ${\rho }_{SHG}$ value, which means that the SHG signal could tend to be zero when the excitation polarization is along the *y*-axis (*b*-axis) of the crystal. Therefore, the polarized SHG imaging technique is a good method to determine the crystal orientation of 2D PdPSe without a polarizer in the signal path, which is unrealizable in MoS_2_^58^. We prepared two samples with different crystal orientations on the same silicon substrate. Sample 1 possesses 6 L, 7 L, 8 L, 9 L and bulk flakes and Sample 2 possesses 6 L and bulk flakes. The *c*-axes of Samples 1 and 2 have an angle of 60° (Fig. 5a). Figure 5b–i show the SHG spatial imaging for different excitation polarization orientations. When the excitation polarization is parallel to the *c*-axis of Sample 1, the SHG signal in Sample 1 shows the maximum intensity. In contrast, the SHG signal in Sample 2 is much weaker than that in Sample 1 (Fig. 5b). Figure 5c displays that when the laser polarization is rotated 10° from the *c*-axis of Sample 1, SHG intensities of Sample 1 (Sample 2) become weaker (stronger), respectively. As the excitation polarization orientation is in the middle of the *c*-axes of Samples 1 and 2, both samples with 6 L show the same SHG intensity (Fig. 5d). When the excitation polarization orientation is closer to the *c*-axis of Sample 2, Sample 1 (Sample 2) show weaker (stronger) SHG intensities (Fig. 5e–g). When the excitation polarization orientation is perpendicular to the *c*-axis of Sample 1, the SHG intensity of Sample 1 shows the minimum value (Fig. 5g), compared with that in Fig. 5b. Furthermore, the SHG intensity of 8 L (C areas) is much weaker than that for 6 L (A areas), which is because 8 L PdPSe has a higher ${\rho }_{SHG}$ value shown in Fig. 4g. It means that this technique can be used to distinguish 4 L, 6 L and thicker layers of even-layer PdPSe in a large area, which cannot be realized in MoS_2_.^58^ Figure 5h displays the SHG spatial imaging result as the excitation polarization orientation is in the middle of *b*-axes of two samples, both samples show the same SHG intensity in 6 L but much smaller than those in Fig. 5d. Finally, as shown in Fig. 5i, when the excitation polarization is along *b*-axis of Sample 2, its SHG intensity shows the minimum value, which is much smaller than that in Fig. 5f, revealing its strong anisotropic SHG. This imaging technique can be used to spatially determine crystal orientation in large crystal areas, and polarization-dependent spatial encoding.

## Discussion

In conclusion, we investigated unique inversion-symmetry-broken behaviors and strong nonlinearities, theoretically and experimentally, in even-layer 2D PdPSe, which are distinctly different from common TMDCs. Through SHG and THG measurements, we determined the second-order susceptibility (${\chi }^{(2)}$) and third-order susceptibility (${\chi }^{(3)}$) to be 64 pm/V and 6.2 × 10^−19^ m^2^/V^2^, respectively. According to Tables S2 and S4, the nonlinear optical susceptibilities are smaller than those in a few of 2D materials (e.g., MoSe_2_) and a few of bulk crystals (e.g., Si), but larger than those in most of 2D materials and commercial nonlinear bulk crystals. Especially, the nonlinear optical susceptibilities are larger than those in mature bulk materials used in the on-chip photonic device (e.g., Si_3_N_4_), which could provide the second-order effect or enhance nonlinear effects of the on-chip photonic device by integrating 2D PdPSe on the device. Importantly, in contrast to MoS_2_ and GaSe without in-plane anisotropy, we showed that 2D PdPSe possesses strong nonlinear optical anisotropy with a maximum ${\rho }_{SHG}$ value of ~45, which is the largest in all atomically layered 2D materials, to the best of our knowledge. The maximum ${\rho }_{THG}$ value of PdPSe crystal is ~7, which is also larger than that in all the 2D layered materials. Furthermore, ${\rho }_{SHG}$ and ${\rho }_{THG}$ values displayed unique wavelength- and layer-dependent behaviors. Specifically, we can harness ${\rho }_{SHG}$ value in a range from 0.12 to 45 in 8 L PdPSe by choosing the excitation wavelength due to the dispersion of ${\chi }^{(2)}$ values. Finally, we performed polarization-dependent SHG imaging, which proved the layer-number-dependent SHG behavior and strong SHG anisotropy properties. The imaging technique can be used to distinguish crystal orientations in a large area and has a potential for achieving polarization-dependent spatial encodings of nonlinear signals. Moreover, the PdPSe crystal possesses the high damage threshold and high air-stability, which makes it be a potential material in practical applications (Note S14). The simultaneous realization of strong nonlinear optical anisotropy and strong nonlinear optical coefficients in 2D PdPSe opens up opportunities for the next-generation miniaturized photonic devices with polarization functions.

## Materials and methods

### Crystal growth

The single crystals of bulk PdPSe were synthesized via a common chemical vapor transport (CVT) method. A total of 0.5 g reactants with molar ratio (Pd:P:Se = 1:1:1) were loaded in a silica tube, which was sealed under a high vacuum condition (<10^−2 ^Pa). Then, the sealed silica tube was put in a two-zone furnace, whose source zone and growth zone were heated to 950 °C and 850 °C within 24 h, respectively. The furnace was kept at the state for 200 h. Subsequently, the furnace was cooled down to room temperature within 100 h. Finally, the shiny bulk crystals of PdPSe were obtained in the sealed silica tube.

### Sample preparation

The PdPSe flakes were exfoliated from the bulk single-crystal using the following process: Bulk PdPSe crystal was first dispensed on the tape. A layer of gold with a thickness of ~120 nm was deposited on the PdPSe crystal by thermal evaporation. A polydimethylsiloxane (PDMS) attached to a glass slide was used to exfoliate PdPSe flakes from bulk crystals. Large flakes with different layers can be obtained due to the affinity between selenium and gold. The gold coated PdPSe flakes were then transfer onto a silicon oxide/fused silica substrate using a home built transferring stage. Finally, the gold layer was removed in KI/I_2_ solution to expose the PdPSe flakes and rinsed by acetone. To prepare samples for polarized SHG imaging, two PdPSe fakes were transferred onto the silicon oxide substrate close to each other with an orientation (*c*-axis) angle of 60°.

### Raman characterization of 2D PdPSe and thickness determination

A WITec CRM200 confocal Raman microscopy system was used to obtain high resolution Raman spectra. A 532 nm laser was used to excite samples with an objective (×100, NA 0.8) and the signal was analyzed by a spectrometer equipped with an air-cooling charge-coupled device (WITec Instruments Corp, Germany). The angle resolved Raman signal was measured by making a polarizer parallel to the polarization of the excitation light. Meanwhile, the thicknesses of exfoliated 2D PdPSe flakes were measured by AFM.

### SHG and THG measurements

A home-built optical setup with both reflective and transmissive configurations was used for SHG and THG measurements. A tunable femtosecond (fs) laser, generated by an OPA (Mango, ~200 fs, 100 kHz) synchronously pumped by a mode-locked 1030-nm fs laser (Amplitude-Yuja, 100 kHz, 400 fs) was utilized as an excitation source. The fs laser passing through a polarizer and a half wavelength plate (HWP) was focused by an objective lens (×50, NA 0.45) to a spot with a diameter of ~2 μm. The transmitted signal light was collected by another objective (×20, NA 0.45) and analyzed by a spectrometer integrated with a cooling charge-coupled device (CCD). For the parallel/perpendicular SHG measurements, we rotated the HWP and the polarizer simultaneously to detect the SHG signal with the polarization parallel/perpendicular to the polarization of incident lights. For measurements of anisotropic THG, *x*- or *y*-components of THG signals were measured by putting a polarizer along *x*- or *y*-axis of the PdPSe crystal, and then we rotated HWP to tune the excitation polarization. We used the Raman spectroscopy technique to distinguish the two in-plane axes (*b*- and *c-*axes) due to its anisotropic crystal orientation. After that, we used a microscopic system to check the direction of the *c*-axis of the PdPSe crystal. Thus, we can make the original fundamental polarization parallel to the *c*-axis of the PdPSe crystal for measurements of polarized SHG and THG processes. All experiments were performed in the air and room temperature environment. For spatial SHG imaging, 980 and 1300-nm lasers were used as excitation sources and the samples were scanned within the area of 80 μm × 80 μm using a high-resolution *X*–*Y* translation stage. The SHG signals were detected by a photomultiplier (PMT, Model: PMT-CR131-Cooling). A HWP was used to rotate excitation polarization to do polarized spatial SHG imaging. The scan duration of 3 h was set to achieve the high-resolution SHG images with a size of 80 μm × 80 μm.

### Supplementary information

Supplemental Material
