# Author Correction: Strong nonlinear optical processes with extraordinary polarization anisotropy in inversion-symmetry broken two-dimensional PdPSe

**DOI:** 10.1038/s41377-024-01522-1

**Published:** 2024-08-12

**Authors:** Song Zhu, Ruihuan Duan, Xiaodong Xu, Fangyuan Sun, Wenduo Chen, Fakun Wang, Siyuan Li, Ming Ye, Xin Zhou, Jinluo Cheng, Yao Wu, Houkun Liang, Junichiro Kono, Xingji Li, Zheng Liu, Qi Jie Wang

**Affiliations:** 1 School of Electrical and Electronic Engineering, Nanyang Technological University, 639798 Singapore, Singapore; 2 School of Material Science and Engineering, Nanyang Technological University, 639798 Singapore, Singapore; 3 CINTRA CNRS/NTU/THALES, UMI 3288, Research Techno Plaza, Nanyang Technological University, 637371 Singapore, Singapore; 4 School of Materials Science and Engineering, Harbin Institute of Technology, 150001 Harbin, China; 5 Department of Chemistry, National University of Singapore, 117543 Singapore, Singapore; 6 GPL Photonics Lab, State Key Laboratory of Applied Optics, Changchun Institute of Optics, Fine Mechanics and Physics, Chinese Academy of Sciences, 130033 Changchun, China; 7 School of Electronics and Information Engineering, Sichuan University, 610064 Chengdu, Sichuan China; 8 School of Physical and Mathematical Sciences, Nanyang Technological University, 637371 Singapore, Singapore; 9 Departments of Electrical and Computer Engineering, Physics and Astronomy, and Materials Science and NanoEngineering, Rice University, Houston, TX USA

**Keywords:** Optical properties and devices, Carbon nanotubes and fullerenes

Correction to: *Light: Science & Applications*

10.1038/s41377-024-01474-6 published online 27 May 2024

There are some changes in the Acknowledgements section of this article^1^. The new Acknowledgements section should be as follows:

This work was supported by the Singapore Ministry of Education (MOET2EP50120-0009), Agency for Science, Technology and Research (A*STAR) (M22K2c0080, R23I0IR041, and A2090b0144), National Medical Research Council (NMRC) (Award number MOH-000927), National Research Foundation Singapore (Award No. NRF-CRP22-2019-0007 and NRF-CRP23-2019-0007), National Research Foundation, Singapore under its AI Singapore Programme (AISG Award No: AISG2-GC-2023-009), and National Research Foundation (Award No. NRF2020-NRF-ISF004-3520).

It is noticed that Fig. 2b contained a typo. The title of the SHG diagram in Fig. 2b should be “SHG”.
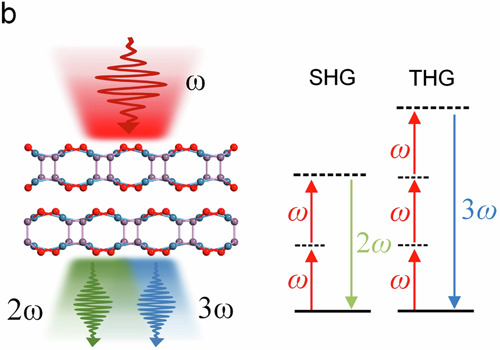


The original article has been corrected.
