# Supplementary material for: Strong nonlinear optical processes with extraordinary polarization anisotropy in inversion-symmetry broken two-dimensional PdPSe
Source: Light Sci Appl. 2024 May 27;13:119. doi: 10.1038/s41377-024-01474-6 (PMC11130276; doi:10.1038/s41377-024-01474-6)
Supplement: Supplementary file 1 — Supplemental Material [file 41377_2024_1474_MOESM1_ESM.docx]

Strong nonlinear optical processes with extraordinary polarization anisotropy in inversion-symmetry broken two-dimensional PdPSe

Song Zhu^1,†^, Ruihuan Duan^2,3,†^, Xiaodong Xu^5,†^, Fangyuan Sun^1^,^†^, Wenduo Chen^1^, Fakun Wang^1^, Siyuan Li^1^, Ming Ye^1^, Xin Zhou^6^, Jinluo Cheng^7^, Yao Wu^2^, Houkun Liang^8^, Junichiro Kono^4,9^, Xingji Li^5^,*, Zheng Liu^2,3^,*, Qi Jie Wang^1,3,4^,*

^1^School of Electrical and Electronic Engineering, Nanyang Technological University, 639798, Singapore

^2^School of Material Science and Engineering, Nanyang Technological University, 639798, Singapore

^3^CINTRA CNRS/NTU/THALES, UMI 3288, Research Techno Plaza, Nanyang Technological University, 637371, Singapore

^4^School of Physical and Mathematical Sciences, Nanyang Technological University, 637371, Singapore

^5^School of Materials Science and Engineering, Harbin Institute of Technology, Harbin

150001, China

^6^Department of Chemistry, National University of Singapore, 117543, Singapore

^7^GPL Photonics Lab, State Key Laboratory of Applied Optics,

Changchun Institute of Optics, Fine Mechanics and Physics,

Chinese Academy of Sciences, Changchun, 130033, China

^8^School of Electronics and Information Engineering, Sichuan University, Chengdu, Sichuan 610064, China

^9^Departments of Electrical and Computer Engineering, Physics and Astronomy, and Materials Science and NanoEngineering, Rice University, Houston, TX, USA

^†^These authors contributed equally to this work

*Corresponding authors: qjwang@ntu.edu.sg, z.liu@ntu.edu.sg and lxj0218@hit.edu.cn

**Table of contents**

**Note S1:** X-ray diffraction (XRD) measurement of the PdPSe crystal

**Note S2:** Energy dispersive analytical X-ray spectroscopy (EDX) measurement of the PdPSe crystal

**Note S3:** Layer dependent Raman spectra of few-layer PdPSe flakes

**Note S4:** Atomic force microscopic (AFM) measurement

**Note S5:** SHG spatial imaging of few-layer PdPSe flakes

**Note S6:** Anisotropic THG processes in the bulk PdPSe

**Table S1:** Evolution of the crystal system with the change of the layer number

**Note S7:** Absorption spectra for different layers

**Note S8.** Bandgap results of PdPSe with different thicknesses

**Table S2:** The comparison between the second-order susceptibility ($\chi^{(2)}$) of 6 L PdPSe to other 2D materials

**Table S3:** The comparison between the SHG anisotropic ratio of few-layer PdPSe to other 2D materials

**Table S4:** The comparison between the third-order susceptibility ($\chi^{(3)}$) of PdPSe to other typical materials

**Table S5.** The comparison between the THG anisotropic ratio of PdPSe to other 2D materials

**Note S9.** Optical setup for nonlinear spatial imaging

**Note S10.** Calculation results of nonlinear optical responses

**Note S11.** Determination of the $\chi^{(2)}$ and $\chi^{(3)}$values

**Note S12.** Polarization-dependent SHG and THG intensities

**Note S13.** First-principles calculations

**Note S14.** The damage threshold and air-stability

**Note S1:** **X-ray diffraction (XRD) measurement of the PdPSe crystal**

The single-crystalline nature is characterized by the X-ray diffraction (XRD) technique. As shown in **Figure S1**, the diffraction pattern is in good agreement with the simulated XRD pattern, demonstrating the high crystal quality of the PdPSe crystal.


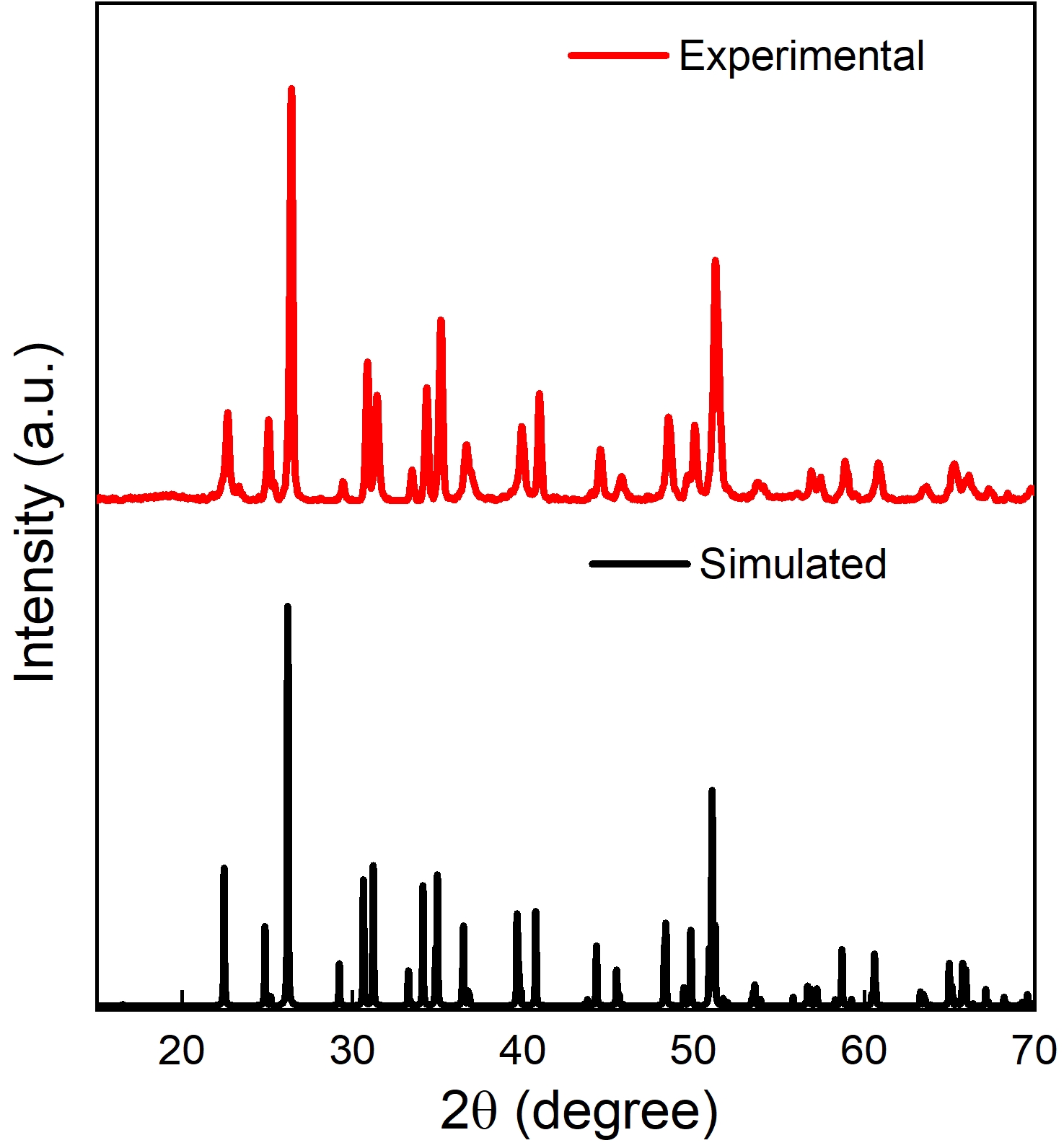


**Figure S1.** Experimental and simulated powder XRD patterns for the PdPSe crystal.

**Note S2: Energy dispersive analytical X-ray spectroscopy (EDX) measurement of the PdPSe crystal**


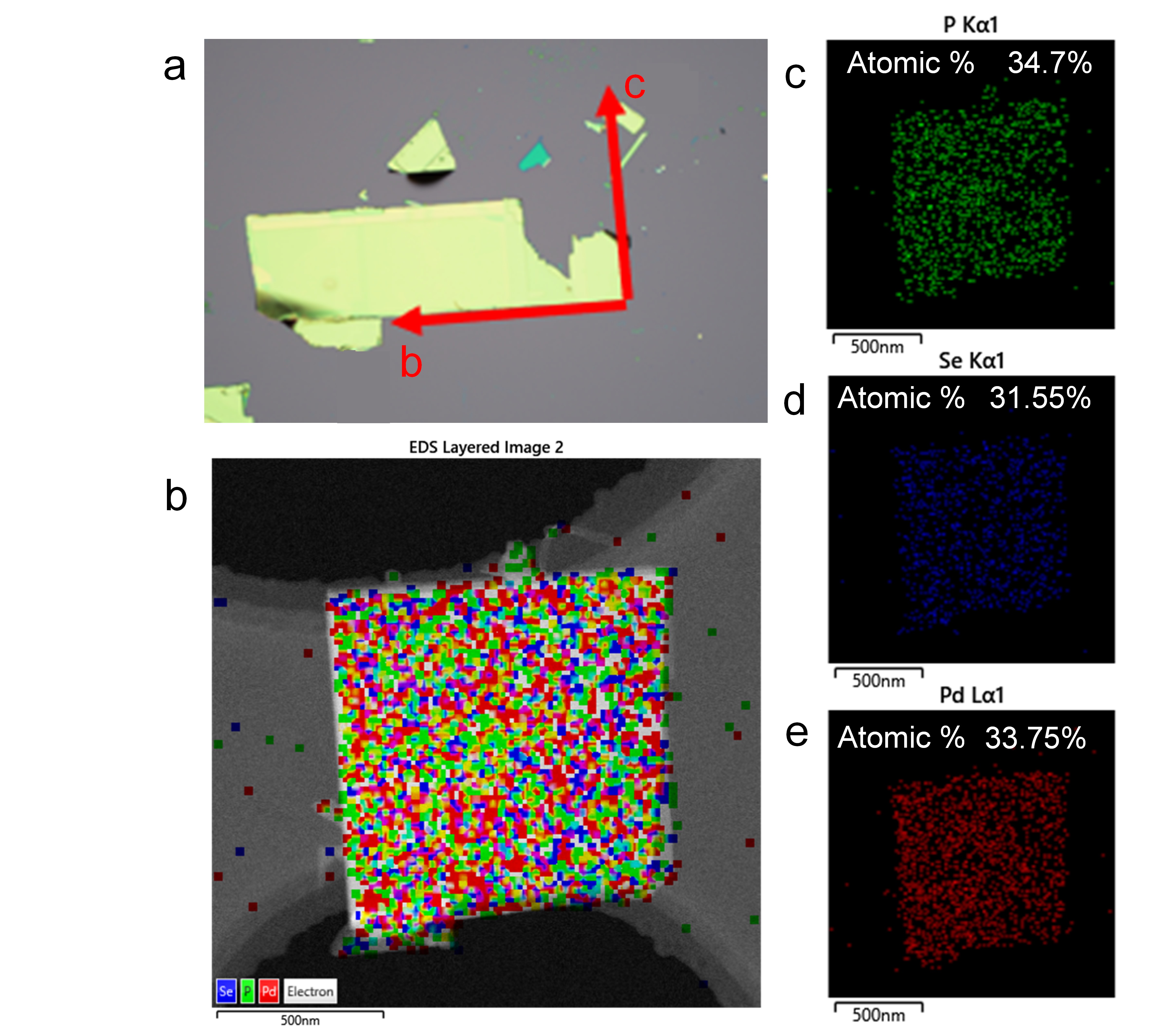


**Figure S2.** Energy dispersive analytical X-ray spectroscopy (EDX) spectra of the PdPSe crystal. (a) Optical microscopic image of bulk PdPSe. (b-e) EDX mappings. The results show that atomic ratio is Pd:P:Se=1.07:1.1:1, demonstrating its good crystal quality.

**Note S3: Layer dependent Raman spectra of few-layer PdPSe flakes**

As shown in **Figure S3**a, 2D PdPSe flakes with many few layers (including 3 L, 4 L, 6 L, 8 L, 9 L, 10 L, 11 L, and bulk) are transferred onto a fused silica substrate. We did not obtain monolayers and bilayer PdPSe flakes due to strong interlayer coupling^1^. **Figure S3**b shows the measured Raman spectra for different layers. There are nine Raman active phonon peaks, namely$B_{1g}^{1}$(120.5 cm^−1^),$B_{3g}$(134.1 cm^−1^),$A_{g}^{1}$(153.6 cm^−1^),$B_{1g}^{2}$(184.6 cm^−1^),$B_{2g}$(217.5 cm^−1^),$A_{g}^{2}$(242.5 cm^−1^),$A_{g}^{3}$(360.9 cm^−1^),$A_{g}^{4}$(389.3 cm^−1^) and$A_{g}^{5}$(472.1 cm^−1^) for bulk PdPSe. The layer-dependent Raman spectra of the PdPSe flakes are also studied. It is found that the major Raman peaks present a little shifts as the thickness decreases from bulk to the thinner layers, which is attributed to the strong interlayer coupling in PdPSe^1^.


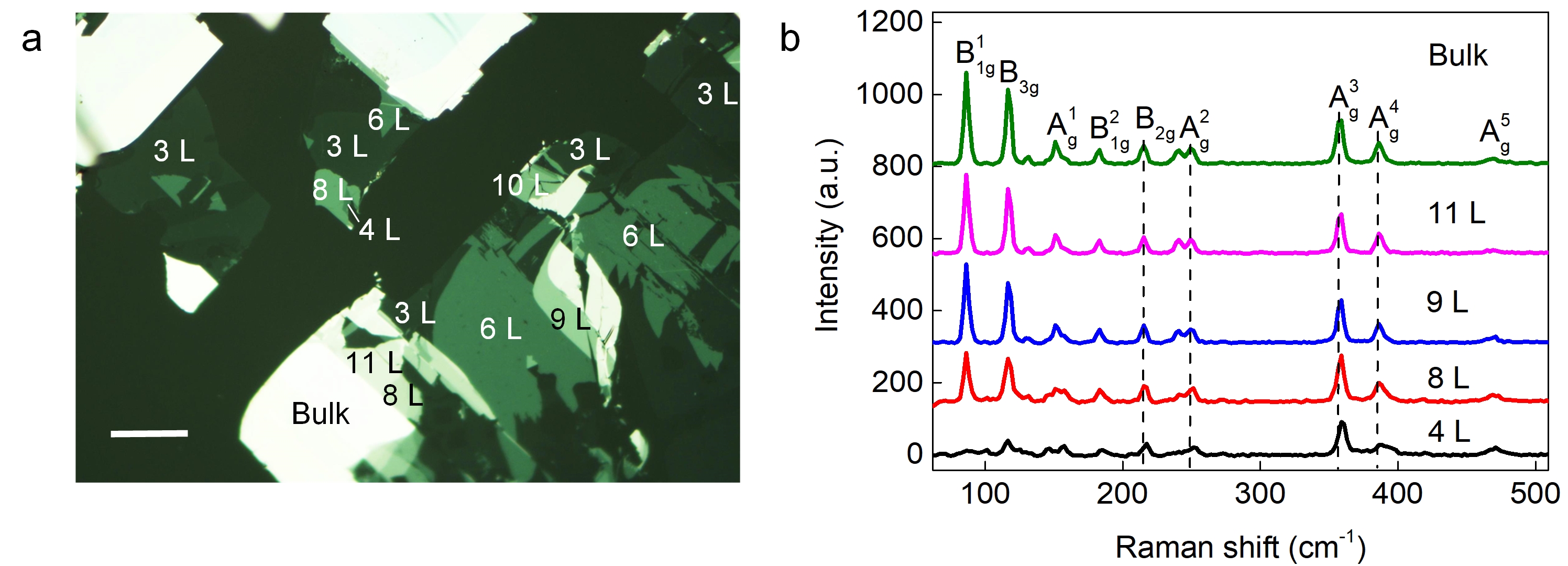


**Figure S3.** (a) The optical microscopic image of a few-layer PdPSe on a fused silica substrate. Scale bar: 10 μm. (b) Raman spectra in different layers.

**Note S4: Atomic force microscopic (AFM) measurement**

As shown in **Figure S4**a, the PdPSe flake includes two parts with different layer numbers. Their thicknesses are confirmed to be 2.2 nm (3 L) and 4.3 nm (6 L) through AFM characterization, as shown in **Figures S4**b and **S4**c. The thickness difference for each layer in 3 L and 6 L is attributed to the measurement deviations of the AFM technique.


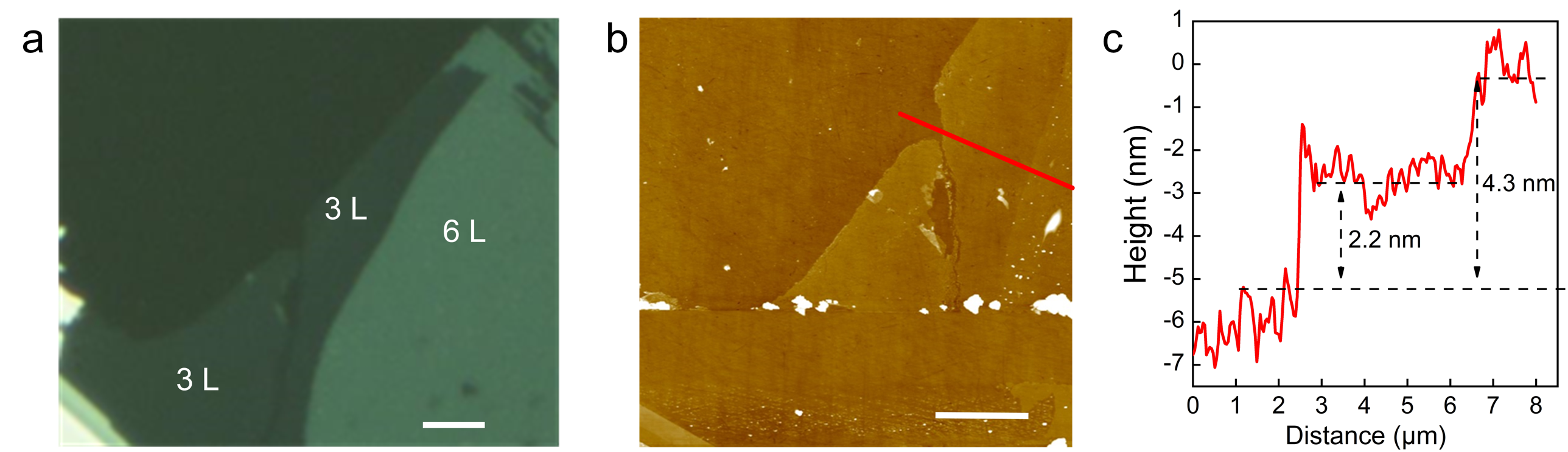


**Figure S4.** (a) Optical microscopic image of few-layer PdPSe flakes. Scale bar: 5 μm. (b) AFM results. Scale bar: 5 μm. (c) Cross-sectional profiles of the edge of the PdPSe flake marked by red line in **Figure S4**b.

**Note S5.** **SHG spatial imaging of few-layer PdPSe flakes**


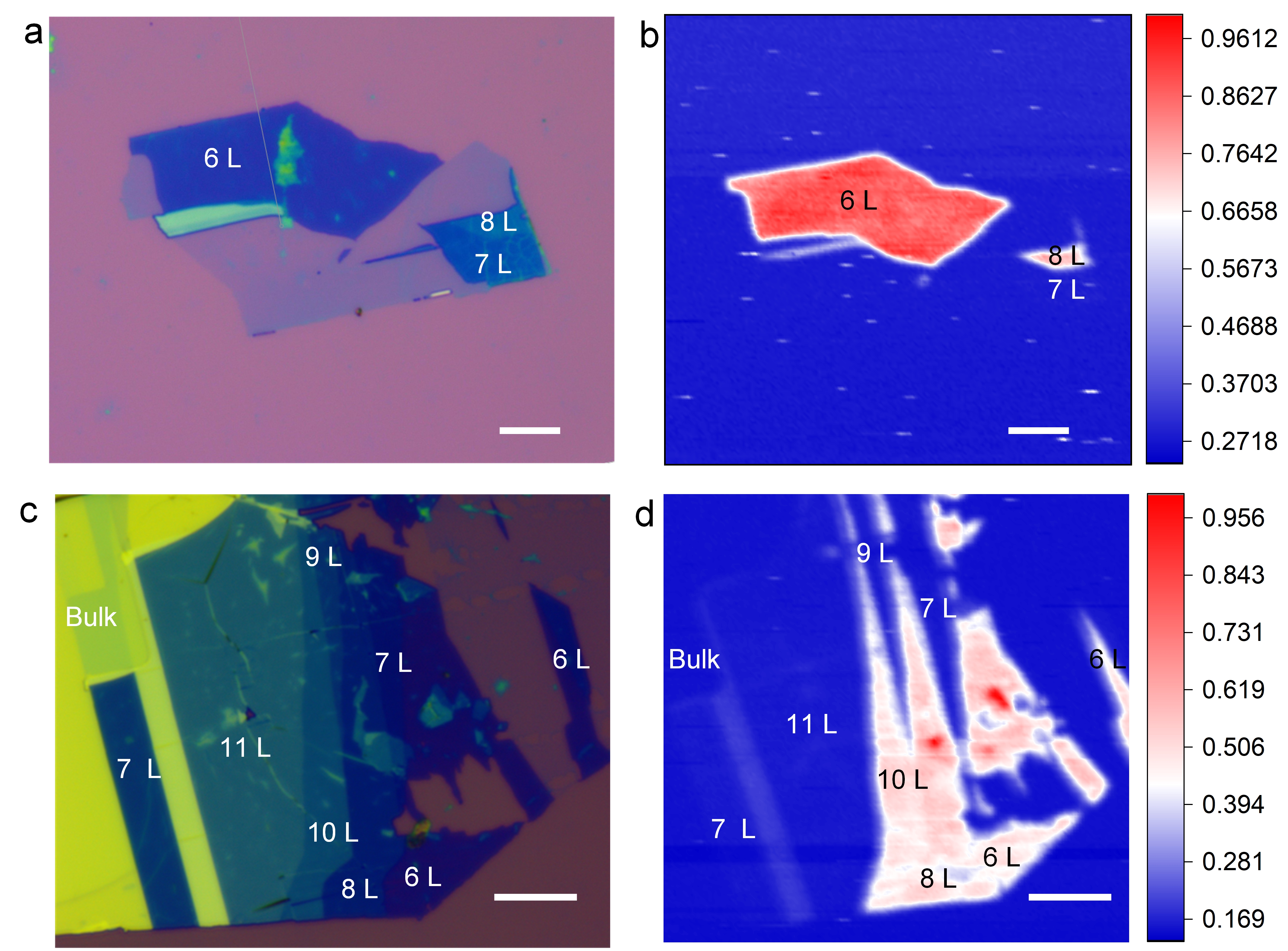


**Figure S5.** SHG spatial imaging of few-layer PdPSe flakes. (a-c) The optical microscopic images of the PdPSe flakes for the SHG imaging. Scale bar: 10 μm. (b-d) The spatial imaging of the SHG signals corresponding to **Figures S5**a and **S5**c, respectively. Scale bar: 10 μm.

To further demonstrate the layer dependent SHG signals of the PdPSe flake, the SHG imaging under 1300-nm excitation is performed. **F****igures S5**a-**S5**c show the scanning areas including 6 L, 7 L, 8 L, 9 L, 10 L, 11 L, and bulk. As shown in **Figures S5**b-**S5**d, the SHG signals can be clearly seen from even layers while are almost negligible in odd layers and the bulk PdPSe. The SHG spatial imaging technique could be used to distinguish even layers from odd layers of large-area PdPSe flakes.

**Note S6.** **Anisotropic THG processes in the bulk PdPSe**

The bulk PdPSe belongs to *mmm* point group, thus its THG susceptibility tensor can be expressed as^2^:

$\chi^{(3)}=\left[\begin{matrix} \chi_{11}^{(3)} & 0 & 0 & 0 & 0 & \chi_{16}^{(3)} & 0 & \chi_{18}^{(3)} & 0 & 0 \\ 0 & \chi_{22}^{(3)} & 0 & \chi_{24}^{(3)} & 0 & 0 & 0 & 0 & \chi_{29}^{(3)} & 0 \\ 0 & 0 & \chi_{33}^{(3)} & 0 & \chi_{35}^{(3)} & 0 & \chi_{37}^{(3)} & 0 & 0 & 0 \end{matrix}\right]$ (S1)

Thus, the *x*- and *y*-polarization components of the generated THG intensity can be expressed as:

$I_{x}^{(3\omega)}\propto\text{(}\chi_{11}^{(3)}\cos^{3}(\theta)+3\chi_{18}^{(3)}\cos(\theta)\sin^{2}(\theta))^{2}$ (S2)

$I_{y}^{(3\omega)}\propto\text{(}\chi_{22}^{(3)}\sin^{3}(\theta)+3\chi_{29}^{(3)}\sin(\theta)\cos^{2}(\theta))^{2}$ (S3)

**Figures S6**a-**S6**b show the optical microscopic image and AFM image of the PdPSe flake, respectively. The thickness of the flake is about 28 nm measured by AFM shown in **Figure S6**c. **Figure S6**d shows polar plots of *x* (red)-and *y* (blue)-components of the THG intensity versus the polarization angle (θ) under 1550-nm excitation. The solid lines are the fittings to the measured data using Equations S2 and S3, which agree well with the measured data. Furthermore, the extracted relative magnitudes of the$\chi^{(3)}$tensor components are$\chi_{11}^{(3)}$:$\chi_{22}^{(3)}$:$\chi_{18}^{(3)}$:$\chi_{29}^{(3)}$=1:0.65:0.2:0.4, which shows its highly anisotropic nonlinear optical response. Furthermore, THG dependent on the elliptical polarization of the excitation light is also studied. The electric field of the excitation beam with the major axis of the polarization ellipsometry is considered as$\overrightarrow{E}=\overrightarrow{e}\pm$, where$\overrightarrow{e}=\overrightarrow{x}\cos(\beta)\pm i\overrightarrow{y}\sin(\beta)$with 𝛽 being the ellipticity angle. Therefore, the electric field of the THG signal can be expressed as:

$E^{(3\omega)}\propto\text{(}\chi_{11}^{(3)}\cos^{3}(\beta)-3\chi_{18}^{(3)}\cos(\beta)\sin^{2}(\beta))\overrightarrow{x}+i(-\chi_{22}^{(3)}\sin^{3}(\beta)+3\chi_{29}^{(3)}\sin(\beta)\cos^{2}(\beta))\overrightarrow{y}$ (S4)

from which the expression of the THG intensity can be expressed as:

$I^{(3\omega)}\propto\text{(}\chi_{11}^{(3)}\cos^{3}(\beta)-3\chi_{18}^{(3)}\cos(\beta)\sin^{2}(\beta))^{2}+(-\chi_{22}^{(3)}\sin^{3}(\beta)+3\chi_{29}^{(3)}\sin(\beta)\cos^{2}(\beta))^{2}$ (S5)

For measurement of elliptical polarization dependent THG, a half waveplate (HWP)


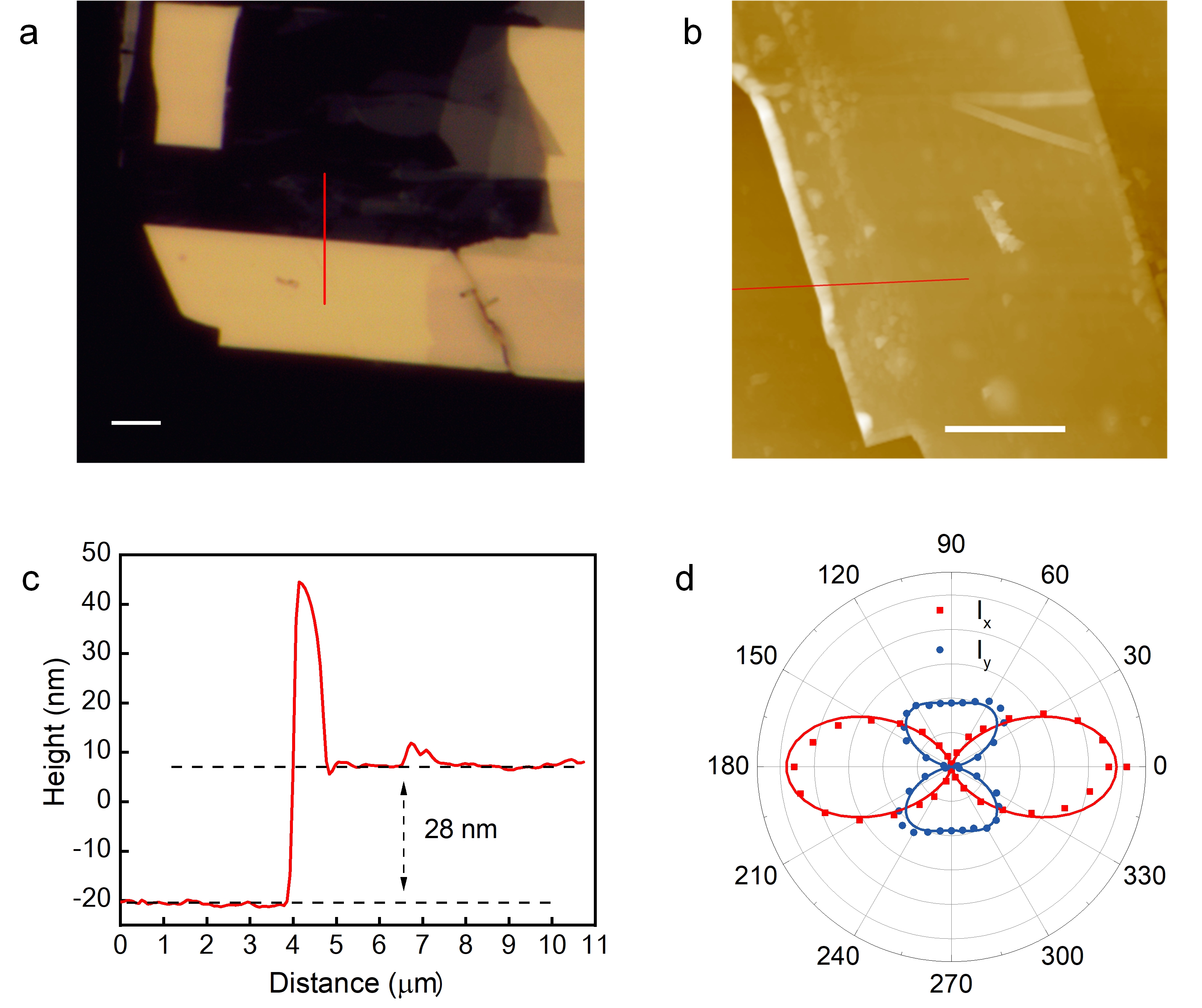


**Figure S6.** THG of the PdPSe flake. a) Optical microscopic image of the bulk PdPSe. Scale bar: 5 μm. (b) The AFM of the PdPSe corresponding to the region in **Figure S6**a. Scale bar: 5 μm. (c) The line profile scanned from the red line in **Figures S6**a and **S6**b. (d) Polar plots of *x* (red)- and *y* (blue)-components of the THG signal versus the polarization angle (θ).

and a quarter waveplate (QWP) are put in front of the sample to generate elliptical light while setting the initial excitation polarization along the *x* (*c*)-axis. The ellipticity of the excitation beam is determined by the rotation angle of the fast axis of the HWP with respect to the initial excitation polarization. We can get the polarization state of excitation beam varying from linear polarization 𝛽=0°+*m*·90° to circular polarization 𝛽=0°+*m*·45°. As shown in **Figure S7**, the THG intensity is the maximum when the sample is excited by the linearly polarized light, while tends to be zero when it is excited by the circular light. Further, the difference in the THG response between *x*- and *y*- excitation shows its highly anisotropic THG.


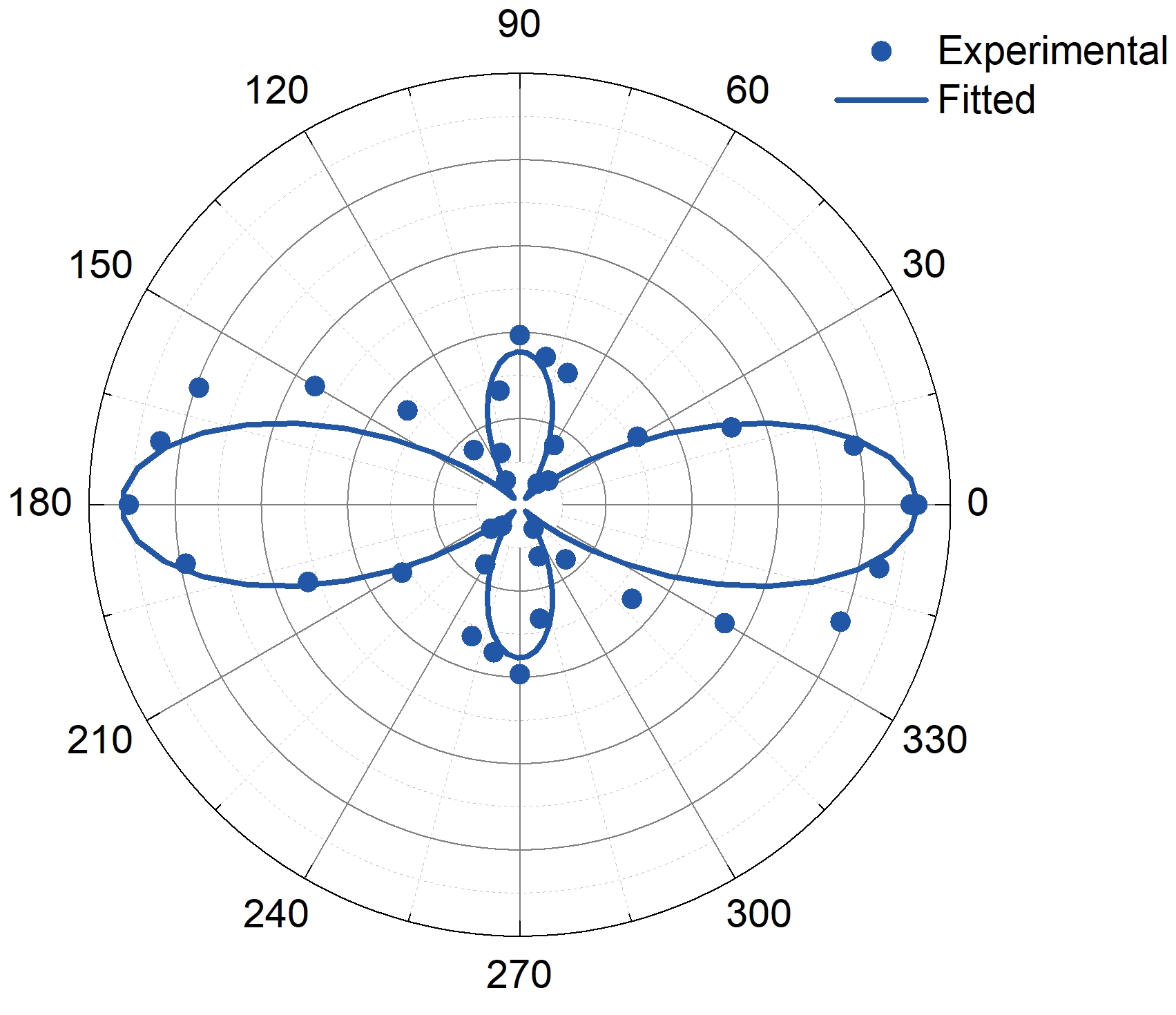


**Figure S7.** Dependence of the THG intensity on the ellipticity angle of the fundamental light. 0° and 180° correspond to the linear polarization along the *c*-axis; 90° and 270° correspond to the linear polarization along the *b*-axis; while 45°, 135°, 225°, and 315° correspond to the circular excitation.

**Table S1. Evolution of the crystal system with the change of the layer number**


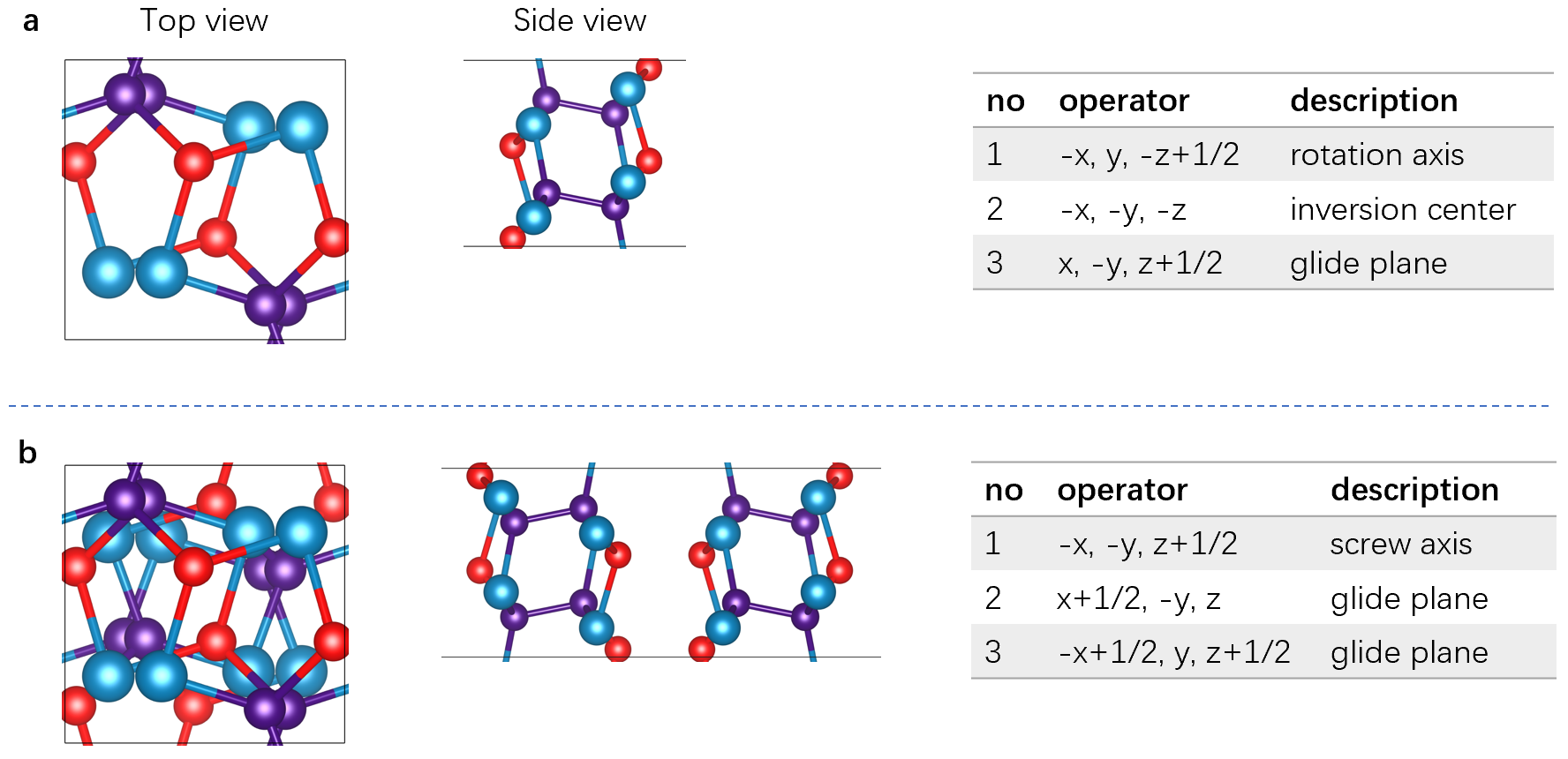


**Figure S8**. Atomic structures and symmetry operators of (a) 1 L and (b) 2 L PdPSe. We analyzed the atomic structure from the view of symmetry, as shown in **Figure S8**. The 1 L with *C_2h_* (2/m) point group has three symmetric operations (one rotation axis, one inversion center and one glide plane) to keep inversion symmetry (**Figure S8**a), while the 2 L with *C_2v_* (mm2) point group has three symmetric operations (one screw axis and two glide planes) breaks the central inversion symmetry (see **Figure S8**b). This broken inversion symmetry results in the nonzero second-order tensile elements in all even-layer PdPSe.

**Note S7. Absorption spectra for different layers**

The differential reflectance spectrum is calculated as: (R-R_0_)/R_0_, which reflects absorption of the sample^3,4^, where R and R_0_ are measured reflectance of the sample and the substrate, respectively. A home-built optical system is used to measure differential reflectance spectra of 3 L, 4 L, and 8 L PdPSe crystals. As shown in **Figure S9**, the absorption of the few-layer PdPSe increases with the layer number, and the absorption peaks are around 490 nm, 620 nm and 770 nm. The peak around 770 nm is derived from the indirect bandgap of PdPSe.


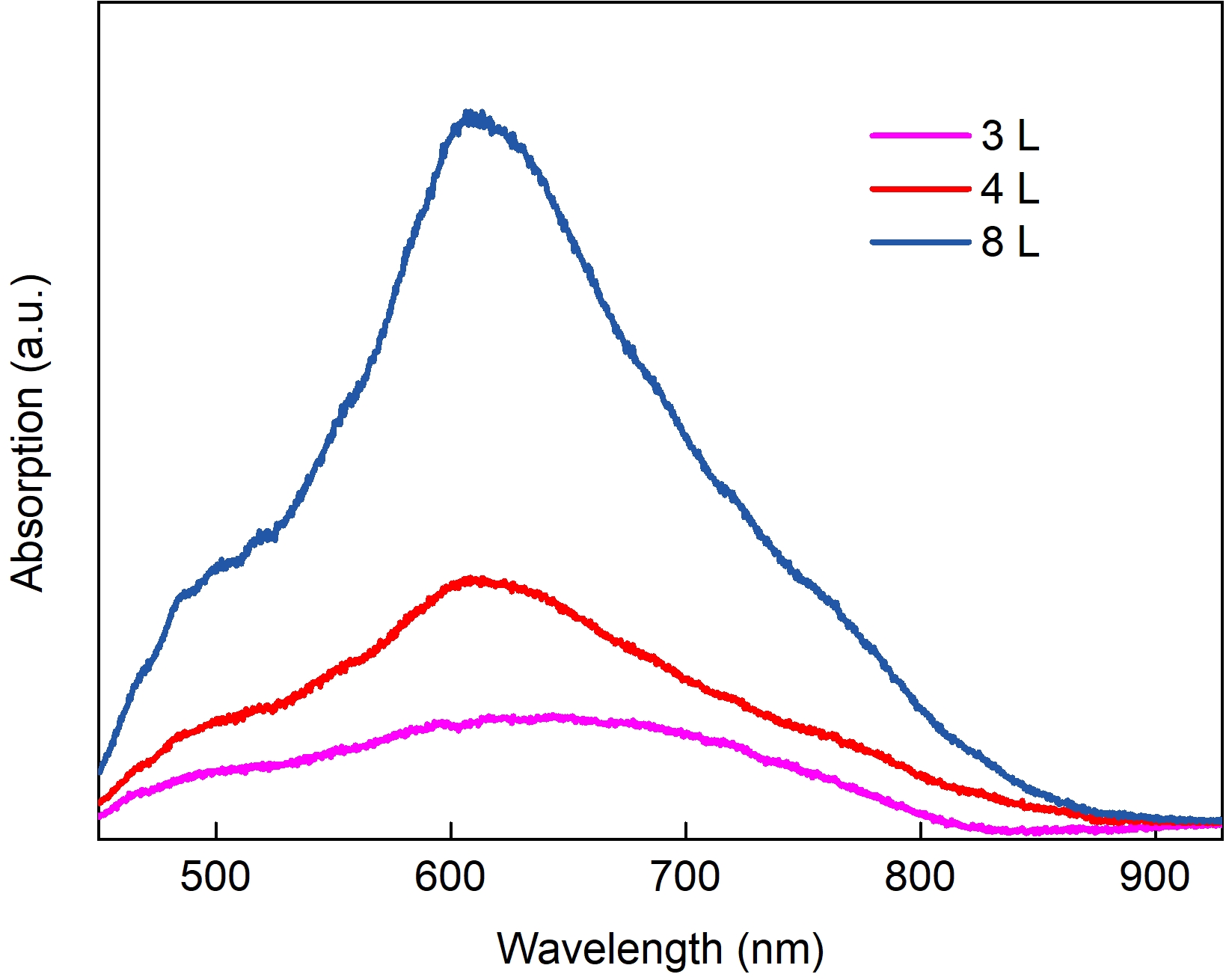


**Figure S9.** Linear absorption spectra of 3 L, 4 L, 8 L PdPSe flakes.

**Note S8. Bandgap results of PdPSe with different thicknesses**





**Figure S10.** Calculated band structure. (a-g) The band structures of PdPSe with different layers. (h) Bandgap versus layer number (L). The red dots in **Figure S10**h are the experimental results (3 L, 4 L, 8 L and bulk PdPSe) based on Tauc plot of (*αhν*)*^1/2^*, where *α* is the absorption coefficient, *hν* is the photon energy. The inset shows the extracted Tauc plot of bulk PdPSe. It can be found that the experimental results are in line with theoretical calculations.

**Table S2. The comparison between the** **second-order susceptibility (**$\chi^{(2)}$**) of 6 L PdPSe to other typical 2D materials and several commercial bulk crystals.**

| Materials | $\chi^{(2)}(pm/V)$ | Excitation conditions | Thickness | Ref. |
| --- | --- | --- | --- | --- |
| **PdPSe** | **64** | **1300 nm, 200 fs, 100 kHz** | **6 L** | **This work** |
| PdSe_2_ | 58 | 800 nm, 80 fs, 80 MHz | 4 L | 5 |
| WS_2_ | 2 | 800 nm, 150 fs, 80 MHz | 1 L | 6 |
| MoS_2_ | 45 | 1300 nm, 30 ps, 50 Hz | 1 L | 7 |
| MoSe_2_ | 100 | 1600 nm, ~ps, 50 Hz | 1 L | 8 |
| ε-InSe | 13 | 800 nm, 150 fs, 80 MHz | 20 nm | 6 |
| GaTe | 1.15 | 1560 nm, 228 fs, 75 MHz | 14 nm | 9 |
| ε-GaSe | 11 | 800 nm, 150 fs, 80 MHz | 20 nm | 6 |
| GaS | 47.98 | 800 nm, 100 fs, 80 MHz | 2 L | 10 |
| NbOI_2_ | 190 | 1050 nm, 50 fs, 1 kHz | 1 L | 11 |
| Nb_3_SeI_7_ | 6.71 | 1064 nm | 20 nm | 12 |
| Quartz | 0.6 | _ | Bulk | 13 |
| LiNbO_3_ | 60 | _ | Bulk | 13 |
| BBO | 4.4 | _ | Bulk | 13 |
| KDP | 0.86 | _ | Bulk | 13 |
| KD_2_PO_4_ | 0.84 | _ | Bulk | 13 |
| LiIO_3_ | 14 | _ | Bulk | 13 |
| GaAs | 340 | _ | Bulk | 14 |

**Table S3. The comparison between the SHG anisotropic ratio of few-layer PdPSe to other 2D materials.**

| Materials | $\rho_{SHG}$ | Excitation conditions | Thickness | Ref. |
| --- | --- | --- | --- | --- |
| **PdPSe** | **15** | **980 nm, 200 fs, 100 kHz** | **4 L** | **This work** |
| **PdPSe** | **16** | **980 nm, 200 fs, 100 kHz** | **6 L** | **This work** |
| **PdPSe** | **45** | **980 nm, 200 fs, 100 kHz** | **8 L** | **This work** |
| MoS_2_ with TiO_2_ nanowires | ~2 | 800 nm | 1 L | 15 |
| ReS_2_ | ~1.8 | 1558 nm, 8.8 ps, 18.5 MHz | 6 L | 16 |
| **ReS_2_** | **3.7** | **980 nm, 200 fs, 100 kHz** | **8 L** | **This work** |
| MoS_2_/CrOCl heterojunction | ~2 | 1024 nm, 1 ps, 40 MHz | 1 L | 17 |
| SnS | 8.64 | 800 nm, 140 fs, 80 MHz | ~11 L | 18 |
| NbOI_2_ | 7.6 | 900 nm, 50 fs, 1 kHz | 20 nm | 11 |
| **NbOI_2_** | **1.5** | **980 nm, 200 fs, 100 kHz** | **8 L** | **This work** |
| PdSe_2_ | 10 | 800 nm | 6 L | 19 |
| **PdSe_2_** | **2.5** | **980 nm, 200 fs, 100 kHz** | **8 L** | **This work** |


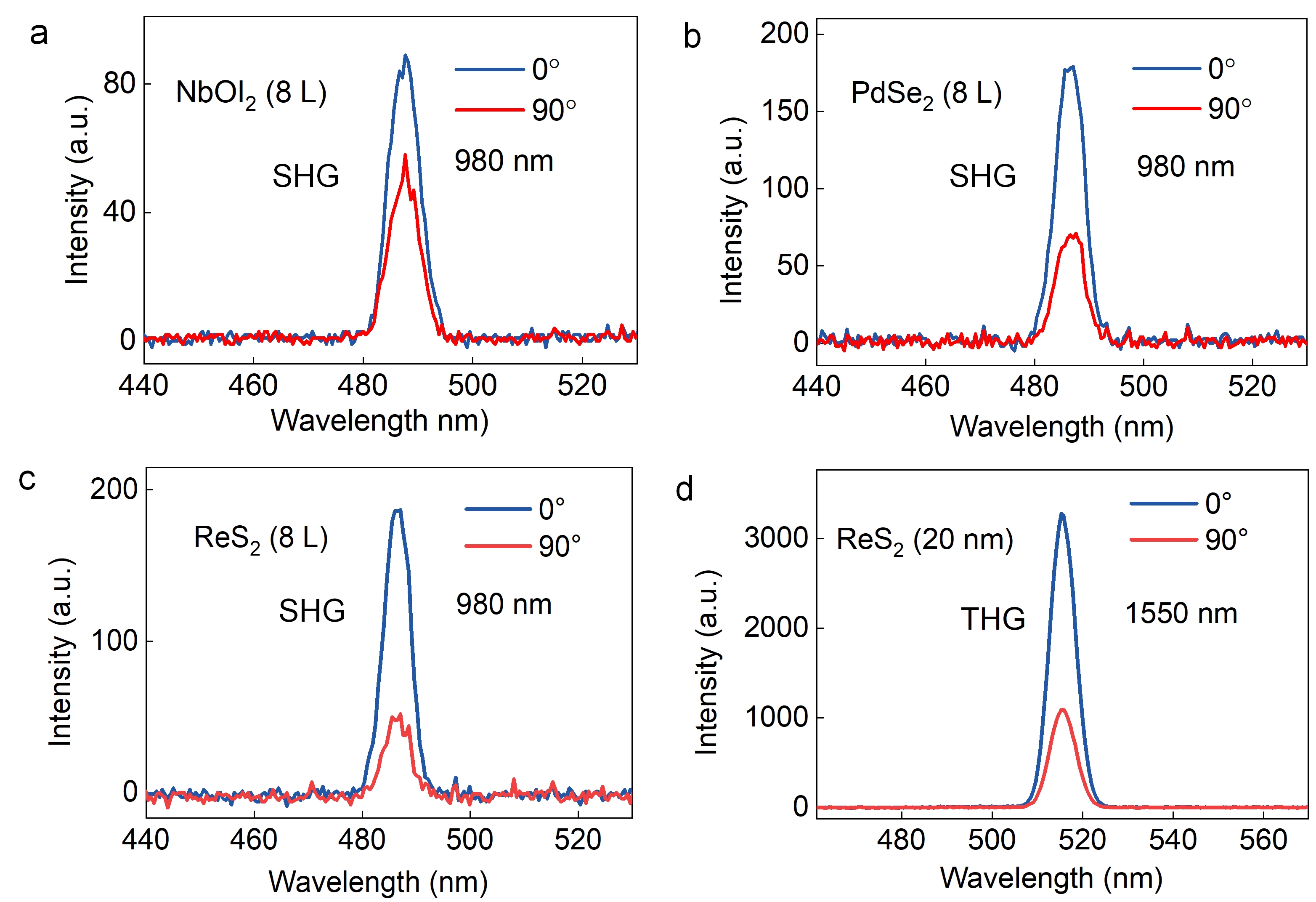


**Figure S11.** (a) SHG spectra of the NbOI_2_ flake along the two in-plane axes. (b) SHG spectra of the PdSe_2_ flake along the two in-plane axes. (c) SHG spectra of the ReS_2_ flake along the two in-plane axes. (d) THG spectra of the ReS_2_ flake along the two in-plane axes.

**Table S4. The comparison between the third-order susceptibility (**$\chi^{(3)}$**) of PdPSe to other typical materials.**

| Materials | $\chi^{(3)}(10^{-19}m^{2}/V^{2})$ | Excitation conditions | Thickness | Ref. |
| --- | --- | --- | --- | --- |
| **PdPSe** | **6.2** | **1550 nm, 200 fs, 100 kHz** | **8 L** | **This work** |
| ReS_2_ | 10 | 1515 nm, 250 fs, 80.54 MHz | Multilayer | 20 |
| MoS_2_ | 3.6 | 1560 nm, 150 fs, 50 MHz | 1 L | 21 |
| WS_2_ | 2.4 | 1560 nm, 150 fs, 50 MHz | 1 L | 21 |
| MoSe_2_ | 2.2 | 1560 nm, 150 fs, 50 MHz | 1 L | 21 |
| WSe_2_ | 1 | 1560 nm, 150 fs, 50 MHz | 1 L | 21 |
| Graphene | 1.5 | 1550 nm, 150 fs, 89 MHz | 1 L | 22 |
| BP | 1.4 | 1550 nm, 200 fs, 35 MHz | 14.5 nm | 23 |
| hBN | 0.084 | 1064 nm, 150 fs, 80 MHz | 60 nm | 24 |
| Si_3_N_4_ | 0.28 | 1064 nm, 70 ps, 1 kHz | Bulk | 25 |
| Fused silica | 0.002 | 1064 nm | Bulk | 26 |
| TiO_2_ | 0.21 | _ | Bulk | 13 |
| LiNbO_3_ | 0.034 | 1064 nm | Bulk | 27 |
| CdS | 0.98 | _ | Bulk | 13 |
| Diamond | 0.025 | _ | Bulk | 13 |
| ZnSe | 0.62 | _ | Bulk | 13 |
| Al_2_O_3_ | 0.0031 | _ | Bulk | 13 |
| As_2_S_3_ glass | 4.1 | _ | Bulk | 13 |
| Si | 1.12×10^6^ | _ | Bulk | 28 |
| Ge | 5.6 | _ | Bulk | 12 |

BP: black phosphorus; hBN: hexagonal boron nitride

**Table S5. The comparison between the THG anisotropic ratio of PdPSe to other 2D materials.**

| Materials | $\rho_{THG}$ | Excitation conditions | Thickness | Ref. |
| --- | --- | --- | --- | --- |
| **PdPSe** | **6** | **1550 nm, 200 fs, 100 kHz** | **10 L** | **This work** |
| **PdPSe** | **7** | **1550 nm, 200 fs, 100 kHz** | **Bulk** | **This work** |
| ReS_2_ | 1.5 | 1515 nm, 250 fs, 80.54 MHz | Multilayer | 20 |
| **ReS_2_** | **3** | **1550 nm, 200 fs, 100 kHz** | **20 nm** | **This work** |
| GeAs | 4 | 1560 nm, 90 fs, 80 MHz | 11 nm | 29 |
| GeSe | 2.75 | 1560 nm, 90 fs, 80 MHz | 160 nm | 30 |
| BP | 2.35 | 1560 nm, 100 fs, 80 MHz | 10 nm | 31 |
| (Pb,Sn)S | 1.35 | 1550 nm, 90 fs, 80 MHz | 61 nm | 32 |
| SiP | 1.93 | 1550 nm, 90 fs, 80 MHz | 10 nm | 33 |
| Lengenbachite | 1.9 | 1560 nm, 90 fs, 80 MHz | 28 nm | 34 |
| Cylindrite | 2.33 | 1560 nm, 90 fs, 80 MHz | 10 nm | 35 |
| Mineral Getchellite | 1.88 | 1560 nm, 90 fs, 80 MHz | 17 nm | 36 |

**Note S9.** **Optical setup for nonlinear spatial imaging**


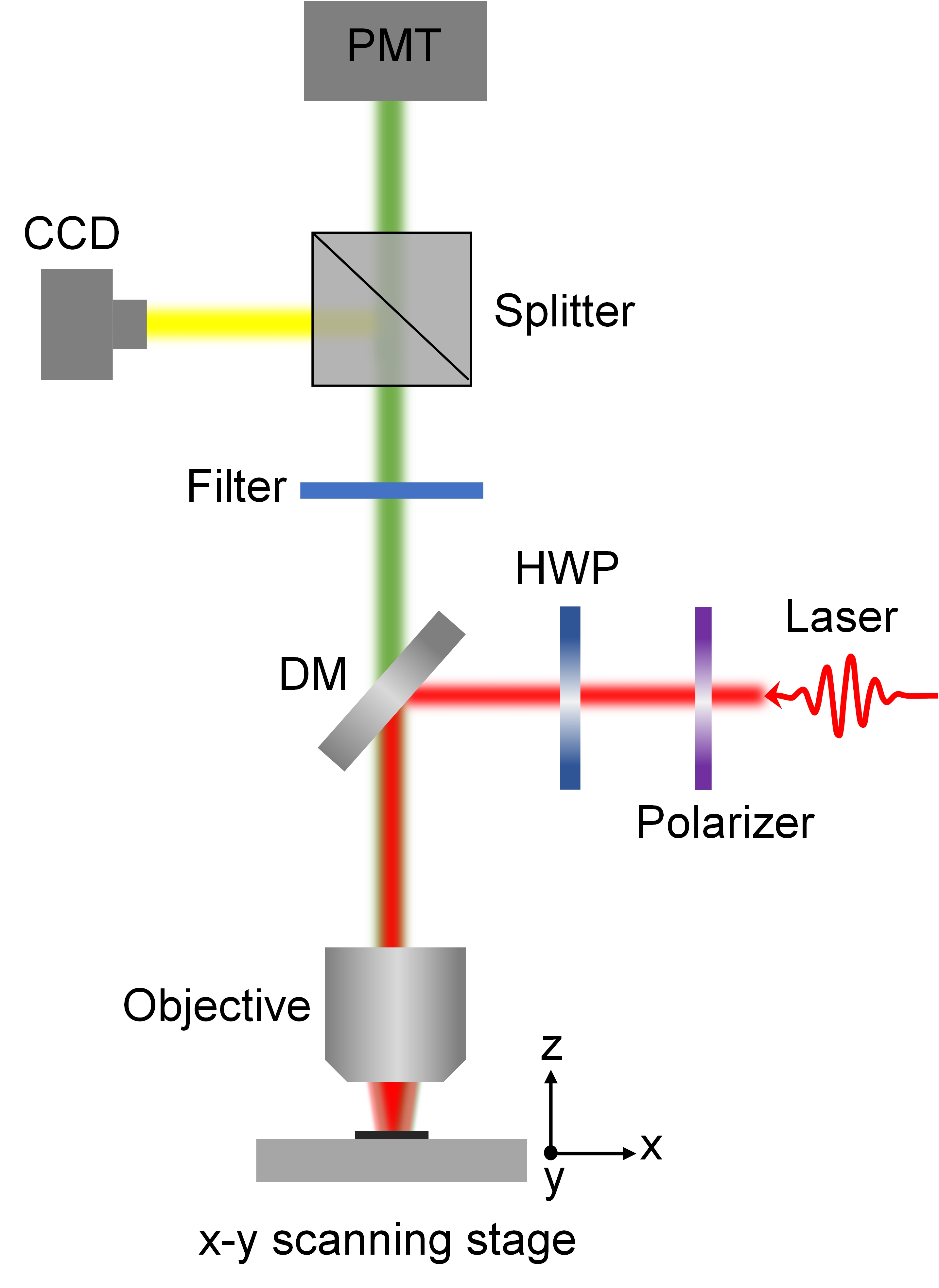


**Figure S12.** Optical setup for nonlinear spatial imaging. HWP: half waveplate; DM: dichromic mirror; PMT: photomultiplier.

**Note S10. Calculation results of nonlinear optical responses**


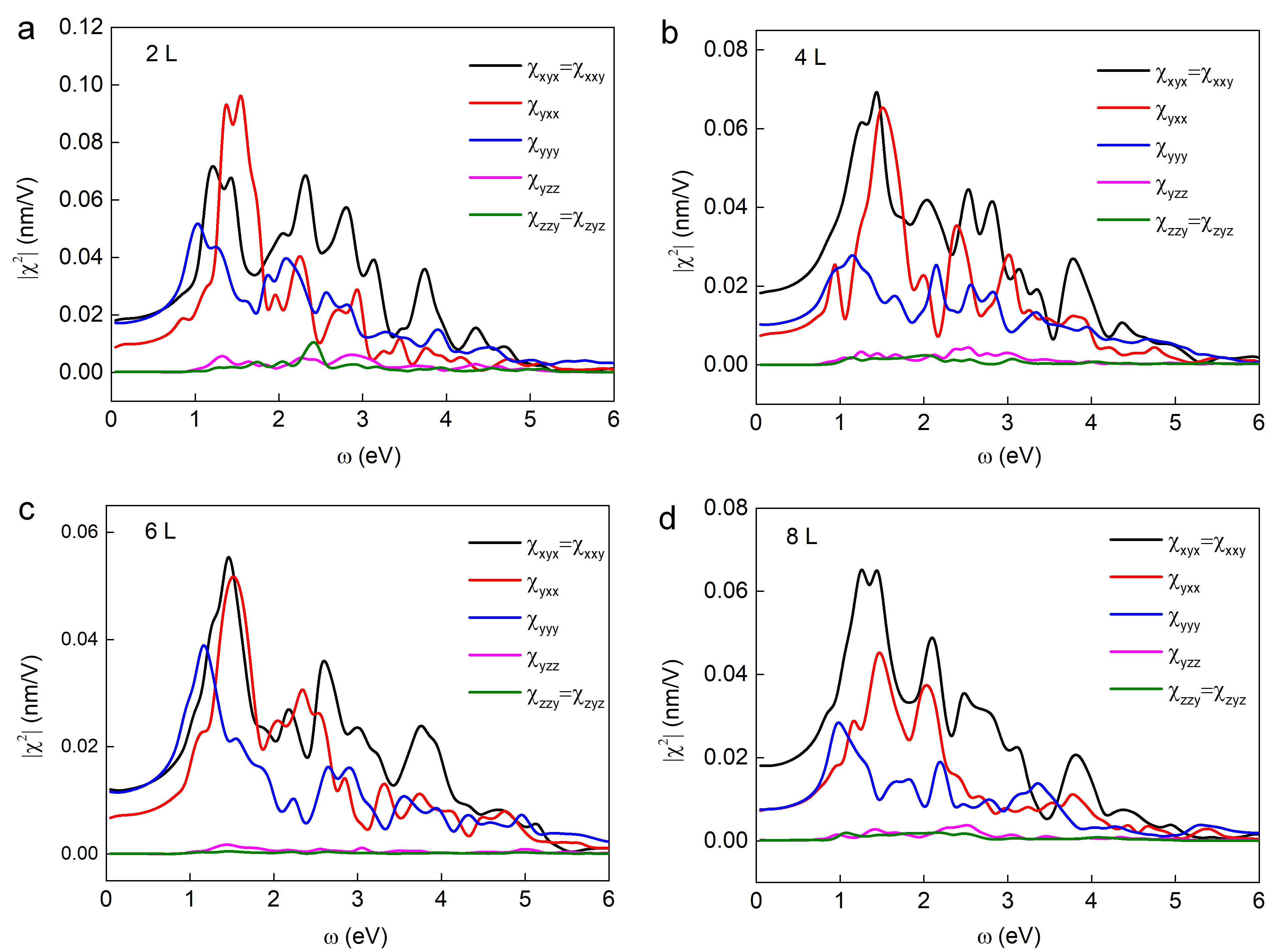


**Figure S13.** SHG magnitude susceptibilities as a function of the fundamental photon energy ($\omega$) in PdPSe with (a) 2 L, (b) 4 L, (c) 6 L, (d) 8 L. **Figure S13** shows the values of SHG tensor elements dependent on the fundamental photon energy. The SHG anisotropic ratio in **Figures 4**f-g is proportional to $\left(\frac{\chi_{yxx}^{(2)}}{\chi_{yyy}^{(2)}}\right)^{2}$.


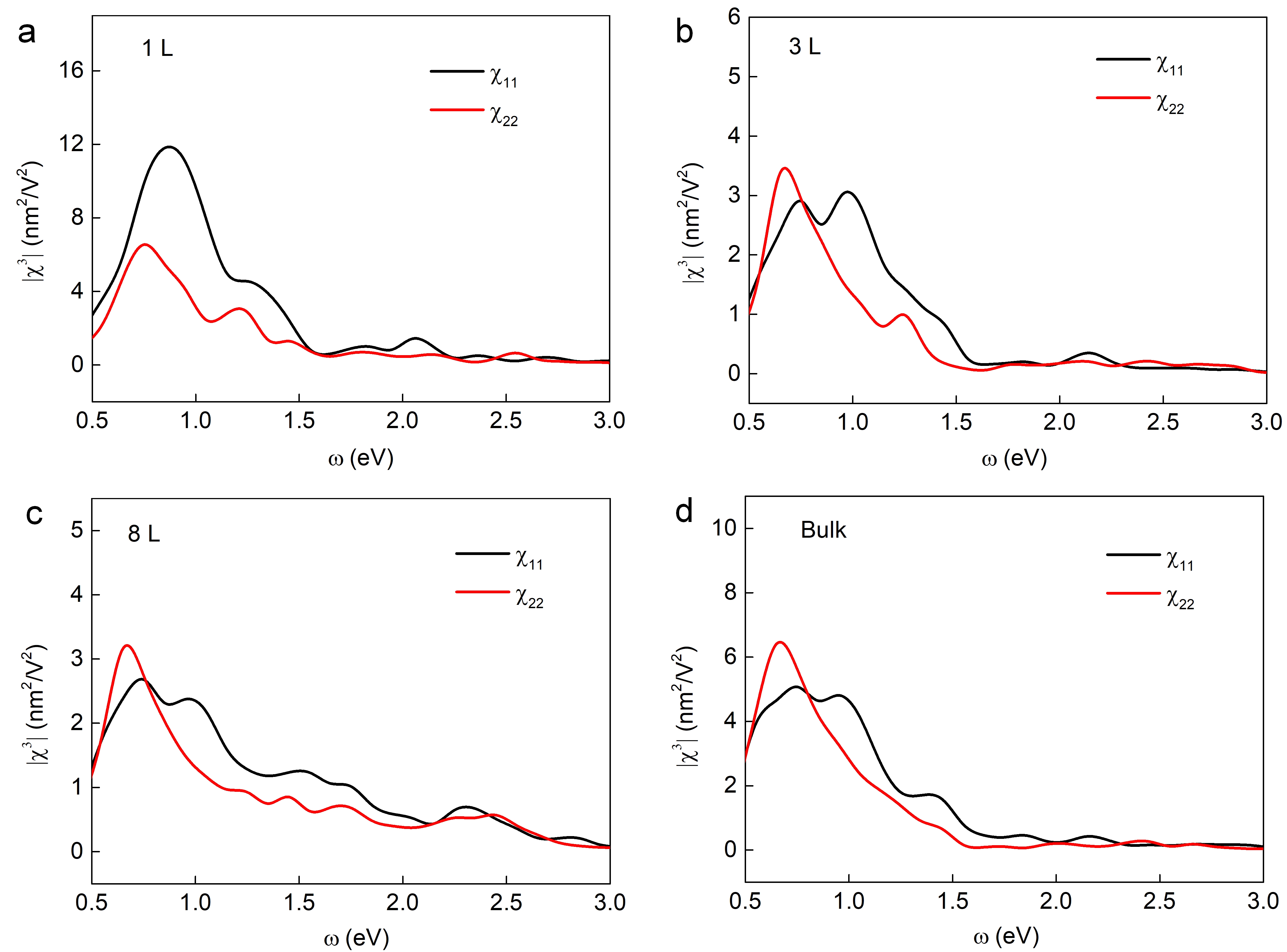


**Figure S14.** THG magnitude susceptibilities as a function of the fundamental photon energy ($\omega$) in PdPSe with (a) 1 L, (b) 3 L, (c) 8 L, and (d) bulk. **Figure S14** shows the values of THG tensor elements dependent on the fundamental photon energy. The THG anisotropic ratio in **Figures 4**f-g is proportional to $\left(\frac{\chi_{11}^{(3)}}{\chi_{22}^{(3)}}\right)^{2}$. The $\chi_{11}^{(3)}$ and $\chi_{22}^{(3)}$correspond to $\chi_{xxxx}^{(3)}$ and $\chi_{yyyy}^{(3)}$, respectively, which is described in Equation S12. In our calculation, we set 100×100×1 k-grid for 2D and 100×100×100 k-grid for 3D system to evaluate the transition for all bands based on TB Hamiltonian and sum all transition probabilities among the k-grid to obtain the nonlinear optical spectra.

**Note S11. Determination of the** $\chi^{(2)}$ **and** $\chi^{(3)}$ **values**

Due to atomic layer thickness of PdPSe, phase-matching in 2D PdPSe can be ruled out when determining its nonlinear susceptibilities^37^. As it is difficult to directly measure powers of the SHG and THG signals from the PdPSe flakes, its second-order and third-order nonlinear susceptibilities can be evaluated by comparing with the well-studied *z*-cut quartz and fused silica. The second-order susceptibility ($\chi_{PdPSe}^{(2)}$) of 6 L PdPSe can be estimated by comparing the measured SHG signals of 6 L PdPSe with that of *z*-cut quartz using the following equation^7^:

$\chi_{PdPSe}^{(2)}=\frac{(n_{_{q}}^{\omega}+1)^{3}}{16\pi d\Delta kn_{_{q}}^{\omega}\sqrt{n_{_{q}}^{2\omega}}}\frac{\lambda}{\lambda_{q}}\sqrt{\frac{I_{q}^{2}(\omega)}{I_{PdPSe}^{2}(\omega)}}\sqrt{\frac{I_{PdPSe}(2\omega)}{I_{q}(2\omega)}F_{q}}\chi_{q}^{(2)}$ (S6)

where$n_{q}^{\omega}$and$n_{q}^{2\omega}$are refractive indexes (RI) of the quartz, *d* is the sample thickness,$\Delta k$is the phase mismatch in the quartz,$\lambda$=1300 nm is the excitation wavelength to the sample,$\lambda_{q}$is the excitation wavelength to the quartz, in which a constructive spectral Maker fringe occurs in the bulk quartz, $I_{P\text{d}PSe}(2\omega)$ is the SHG intensity from the sample (6 L), $I_{q}(2\omega)$ is the SHG intensity from the quartz,$F_{q}$is the Fresnel coefficient of the quartz, which is used to compensate reflection losses, $\chi_{q}^{(2)}$=0.6 pm/V is the second-order susceptibility ($\chi_{q}^{(2)}$) of the quartz. Since the fundamental polarization is parallel to the *c*-axis, the tensor element $\chi_{yxx}^{(2)}$ is dominated. Therefore, according to Equation S6, the $\chi_{yxx}^{(2)}$ value of 6 L PdPSe is estimated to be ~64 pm/V at 1300 nm. To make sure this approach is valid, we also measured the well-known monolayer MoS_2_ through this approach at 1300 nm. As shown in **Figure S15**, the $\chi^{(2)}$ value of monolayer MoS_2_ is calculated to be 60 pm/V, which is comparable to the reported values^7^, verifying validity of this approach.


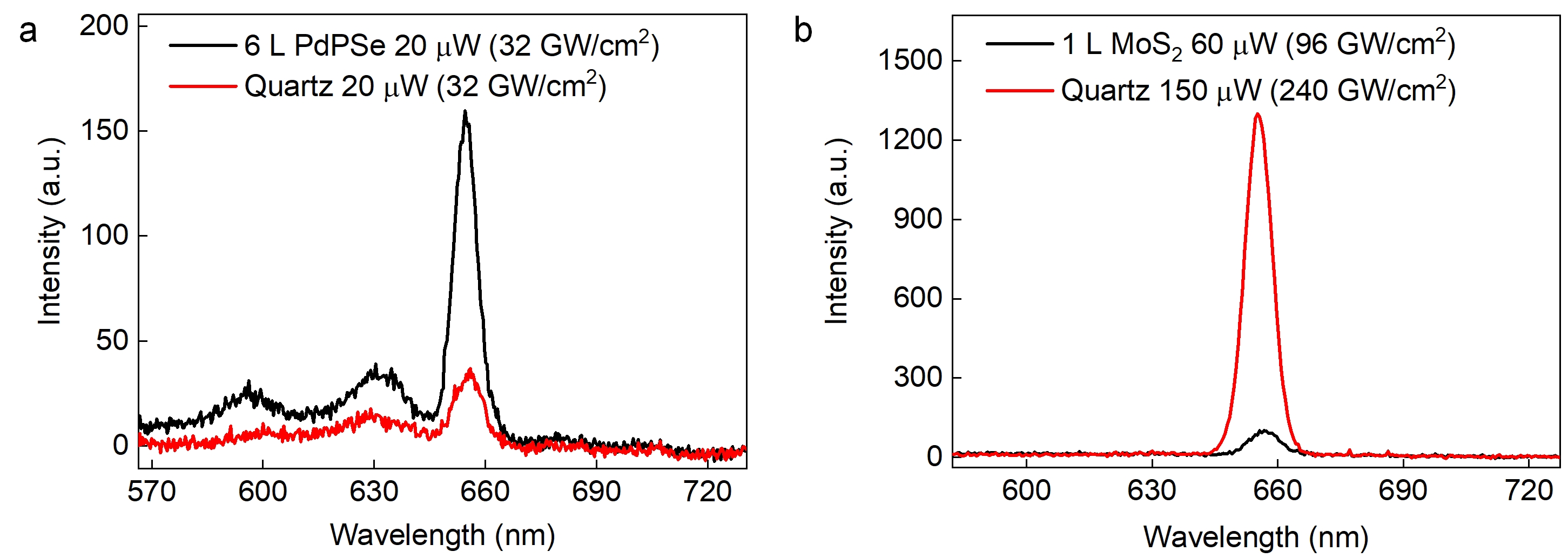


**Figure S15**. (a) SHG spectra of 6 L PdPSe and quartz. (b) SHG spectra of monolayer MoS_2_ and quartz.

When the fundamental polarization is parallel to the *c*-axis, the tensor element $\chi_{11}^{(3)}$ is dominated. The third-order susceptibility ($\chi_{PdPSe}^{(3)}$) can be estimated by comparing the measured THG signal of PdPSe to that of the fused silica substrate using the following equation^24^:

$\chi_{PdPSe}^{(3)}=\frac{\lambda}{6\pi d(n_{s}^{\omega}+n_{s}^{3\omega})}\sqrt{\frac{I_{s}^{3}(\omega)}{I_{PdPSe}^{3}(\omega)}}\sqrt{\frac{I_{PdPSe}(3\omega)}{I_{s}(3\omega)}}\chi_{s}^{(3)}$ (S7)

where$\lambda$is the fundamental wavelength, *d* is the sample thickness,$n_{s}^{\omega}$=1.44, and $n_{s}^{3\omega}$=1.46 are RI of fused silica^38^, $I_{s}(\omega)$and$I_{s}(3\omega)$are the excitation and THG powers of the fused silica substrate, $I_{PdPSe}(\omega)$and$I_{PdPSe}(3\omega)$are the excitation and THG powers of PdPSe, $\chi_{s}^{(3)}$=2.2×10^-22^ m^2^/V^2^ is the thrid-order nonlinear susceptibility of fused silica. the$\chi_{11}^{(2)}$value of PdPSe is estimated to be ~4×10^-19^ m^2^/V^2^ according to Equation S7. We also estimate $\chi_{PdPSe}^{(3)}$ by comparing to monolayer WS_2_ using equation^39^: $\chi_{11}^{(2)}=\frac{d_{WS_{2}}}{d_{PdPSe}}\sqrt{\frac{I_{PdPSe}}{I_{WS_{2}}}}\chi_{WS_{2}}^{(3)}$ and obtained a similar value of ~6.2×10^-19^ m^2^/V^2^. To make sure this approach is valid, we also measured the well-known monolayer MoS_2_ at 1550 nm. A shown in **Figure S16**, the $\chi^{(3)}$ value of monolayer MoS_2_ is calculated to be 4.5×10^-19^ m^2^/V^2^, which is comparable to the reported values^40^, verifying validity of this approach.


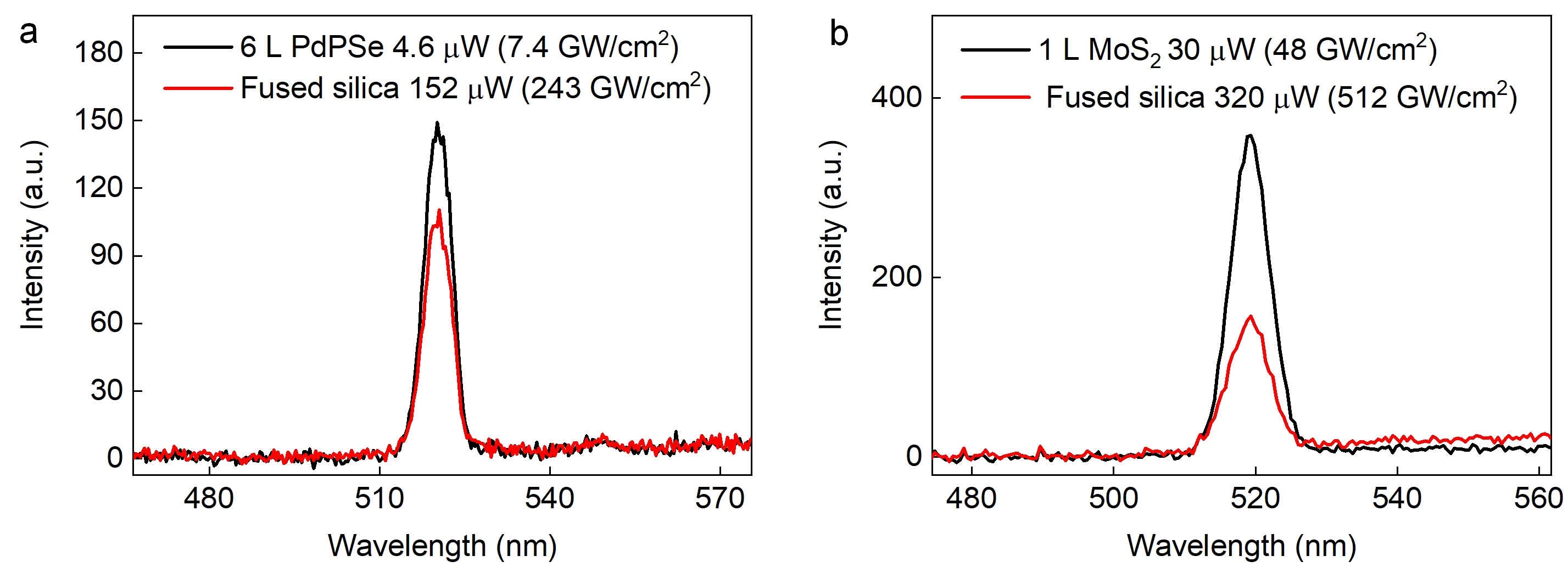
 **Figure S16**. (a) THG spectra of 6 L PdPSe and fused silica. (b) THG spectra of monolayer MoS_2_ and fused silica.

In this work, we used an objective with NA=0.45 for light focusing, which corresponds to a maximum angle of incidence of ~27 degrees in air, or approximately ~10 degrees or smaller in the PdPSe volume, considering to refractive index of the PdPSe crystal. Thus, the ratio of the in-plane and out-of-plane components of the incident light can be roughly estimated as *E_x_*/*E_z_* ∼6 or larger. For $\chi^{(3)}$ tensor elements, if we assume that all the susceptibility components are comparable to each other, the contribution to THG signal from the out-plane component (*E_z_*) will be at least 36 times smaller than the contribution from in-plane components (the case of $\chi_{^{zyyz}}^{(3)}$). On the other hand, the measured $\chi^{(3)}$value of MoS_2_ in our work is 4.5×10^-19^ m^2^/V^2^ using the objective with NA=0.45, verifying that the objective with NA=0.45 is valid to measure THG. For SHG measurement, apart from the measured $\chi^{(2)}$value of MoS_2_, the SHG tensor elements related to *E_z_* ($\chi_{yzz}$, $\chi_{zzy}$ and $\chi_{zyz}$) are much smaller than the other tensor elements (**Figure S13**). Thus, the out-plane component (*E_z_*) could be neglected when we measure SHG even though we use an objective with NA=0.45. Therefore, it is feasible to measure SHG and THG use an objective with NA=0.45.

**Note S12. Polarization-dependent SHG and THG intensities**

As shown in **Figure 1**a, in the *x-y-z* coordinate, *x-*, *y-* and *z*- axes are along *c-*, *b-* and *a*-axes respectively. Since the propagation direction of the fundamental light is normal to the surface of PdPSe, the electric field intensity of the incidence light can be expressed as$\overrightarrow{E}=E_{x}\overrightarrow{x}+E_{y}\overrightarrow{y}=E_{0}\cos(\theta)\overrightarrow{x}+E_{0}\sin(\theta)\overrightarrow{y}$, where$E_{0}$is the electric field intensity of the excitation light, and *θ* represents the angle between the excitation polarization orientation and the *x*-axis (*c*-axis) of the crystal lattice of PdPSe. SHG susceptibility tensor of the inversion symmetry broken PdPSe can be expressed as^13^:

$\chi^{(2)}=\left[\begin{matrix} 0 & 0 & 0 & 0 & 0 & \chi_{xxy}^{\text{（}2\text{）}} \\ \chi_{yxx}^{\text{（}2\text{）}} & \chi_{yyy}^{\text{（}2\text{）}} & \chi_{yzz}^{\text{（}2\text{）}} & 0 & 0 & 0 \\ 0 & 0 & 0 & \chi_{zyz}^{\text{（}2\text{）}} & 0 & 0 \end{matrix}\right]$ (S8)

Therefore, the second-order nonlinear polarization component in the PdPSe crystal is:

$P^{(2\omega)}=\left[\begin{matrix} P_{x}^{(2\omega)} \\ P_{y}^{(2\omega)} \\ P_{z}^{(2\omega)} \end{matrix}\right]=\varepsilon_{0}E_{0}^{2}\left[\begin{matrix} 2\chi_{xxy}^{(2)}\cos(\theta)\sin(\theta) \\ \chi_{yxx}^{(2)}\cos^{2}(\theta)+\chi_{yyy}^{(2)}\sin^{2}(\theta) \\ 0 \end{matrix}\right]$ (S9)

Thus, the *x*- and *y*- polarization components of the generated SHG intensity can be expressed as:

$I_{x}^{(2\omega)}\propto\text{(}2\chi_{xxy}^{(2)}\cos(\theta)\sin(\theta))^{2}$ (S10)

$I_{y}^{(2\omega)}\propto\text{(}\chi_{yxx}^{(2)}\cos^{2}(\theta)+\chi_{yyy}^{(2)}\sin^{2}(\theta))^{2}$ (S11)

THG susceptibility tensor of the inversion symmetry broken PdPSe can be expressed as^2,13^:

$\chi^{(3)}=\left[\begin{matrix} \chi_{11}^{(3)} & 0 & 0 & 0 & 0 & \chi_{16}^{(3)} & 0 & \chi_{18}^{(3)} & 0 & 0 \\ 0 & \chi_{22}^{(3)} & 0 & \chi_{24}^{(3)} & 0 & 0 & 0 & 0 & \chi_{29}^{(3)} & 0 \\ 0 & 0 & \chi_{33}^{(3)} & 0 & \chi_{35}^{(3)} & 0 & \chi_{37}^{(3)} & 0 & 0 & 0 \end{matrix}\right]$ (S12)

where the first subscript 1, 2, 3 refers to *x*, *y*, *z*, respectively, and the second subscript refers to the following:

$$\begin{matrix} xxx & yyy & zzz & yzz & yyz & xzz & xxz & xyy & xxy & xyz \\ 1 & 2 & 3 & 4 & 5 & 6 & 7 & 8 & 9 & 0 \end{matrix}$$

Therefore, the third-order nonlinear polarization component in the PdPSe crystal is:

$P^{(3\omega)}=\left[\begin{matrix} P_{x}^{(3\omega)} \\ P_{y}^{(3\omega)} \\ P_{z}^{(3\omega)} \end{matrix}\right]=\varepsilon_{0}E_{0}^{3}\left[\begin{matrix} \chi_{11}^{(3)}\cos^{3}(\theta)+3\chi_{18}^{(3)}\cos(\theta)\sin^{2}(\theta) \\ \chi_{22}^{(3)}\sin^{3}(\theta)+3\chi_{29}^{(3)}\sin(\theta)\cos^{2}(\theta) \\ 0 \end{matrix}\right]$ (S13)

Thus, the *x*- and *y*-polarization components of the generated SHG intensity can be expressed as:

$I_{x}^{(3\omega)}\propto\text{(}\chi_{11}^{(3)}\cos^{3}(\theta)+3\chi_{18}^{(3)}\cos(\theta)\sin^{2}(\theta))^{2}$ (S14)

$I_{y}^{(3\omega)}\propto\text{(}\chi_{22}^{(3)}\sin^{3}(\theta)+3\chi_{29}^{(3)}\sin(\theta)\cos^{2}(\theta))^{2}$ (S15)

**Note S13. First-principles calculations**

The simulations of nonlinear optical responses in PdPSe were performed using DFT in combination with the Wannier interpolation method. In DFT calculation, the strongly constrained and appropriately normed (SCAN) meta-generalized gradient approximation (METAGGA) exchange-correlation potential^41^ implemented in VASP package^42^ was employed for structural relaxation with a criterion of 0.01 eV/ Å. The lattice parameters were determined to be *a*=13.71 Å, *b*=5.81 Å and *c*=5.84 Å in a good agreement with experimental measurement.^1^ SCAN+rVV10^43^ scheme was chosen to accurately evaluate the weak van der Waals interaction among layers. To reduce the computational cost, the SCAN+U method was adopted to calculate the ground state information of bulk and layered PdPSe. After benchmarking with the experimental band gap of bulk PdPSe, the Hubbard on-site energy U on Pd d-orbitals was determined to be 7 eV. A Gamma-centered *k*-point meshes of 11×11×4 and 11×11×1 were set for bulk and layered PdPSe, respectively. A cutoff energy of 500 eV was set for all DFT calculations. Using maximally localized Wannier functions, the Hamiltonian of tight-binding Hamiltonian was constructed using Wannier90 package^44^ to reproduce the band structure of DFT.

Based on the Hamiltonian constructed by Wannier functions, we further calculated the SHG and THG processes of layered PdPSe. In principle, $\chi^{(2)}$ is composed of the interband contributions ($\chi_{e}^{(2)}$) and mixed interband and intraband contributions ($\chi_{i}^{(2)}$)^44^:

$\chi^{(2)}=\chi_{e}^{(2)}+\chi_{i}^{(2)}$ (S16)

Alternatively, the interband contribution is described as:

$\chi_{e}^{(2)}=\frac{iC}{\omega_{2}m}\sum_{n,m,l,\mathbf{k}} \frac{p_{mn}^{c}}{\omega_{nm}-\omega_{2}}\left(\frac{r_{nl}^{b}r_{lm}^{a}f_{ml}}{\omega_{lm}-\omega_{1}}-\frac{r_{nl}^{a}r_{lm}^{b}f_{ln}}{\omega_{nl}-\omega_{1}}\right)$ (S17)

The mixed contribution is described as:

$\chi_{i}^{(2)}=\frac{-C}{\omega_{2}m}\sum_{n,m,\mathbf{k}} \frac{p_{mn}^{c}}{\omega_{nm}-\omega_{2}}\left(\frac{r_{nm}^{a}f_{mn}}{\omega_{nm}-\omega_{1}}\right)_{;k^{b}}$ (S18)

where with *K* accounting for the usual factors, *r* is the position operator, $\omega_{mn}$is the frequency difference between band *m* and *n*, *f* is the Fermi-Dirac distribution functions, *p* is the polarization operator.

For the third-order optical response, $\chi^{(3)}$ is composed of interband contribution ($\chi_{\chi}^{(3)}$) and intraband contribution ($\chi_{\sigma}^{(3)}$)^44^:

$\chi^{(3)}=\chi_{\chi}^{(3)}+\chi_{\sigma}^{(3)}$ (S19)

Alternatively, the interband contribution is described as:

$\begin{matrix} \frac{\chi_{\chi}^{(3)}}{C}=\sum_{l,m,n,p,\mathbf{k}} \frac{r_{mn}^{d}}{\omega_{nm}-\omega_{3}}\left[\frac{r_{nl}^{c}}{\omega_{lm}-\omega_{2}}\left(\frac{r_{lp}^{b}r_{pm}^{a}f_{mp}}{\omega_{pm}-\omega_{1}}-\frac{r_{lp}^{a}r_{pm}^{b}f_{pl}}{\omega_{lp}-\omega_{1}}\right)-\frac{r_{pm}^{c}}{\omega_{np}-\omega_{2}}\left(\frac{r_{nl}^{b}r_{lp}^{a}f_{pl}}{\omega_{lp}-\omega_{1}}-\frac{r_{nl}^{a}r_{lp}^{b}f_{ln}}{\omega_{nl}-\omega_{1}}\right)\right] \\ \begin{matrix} & & +i \end{matrix}\sum_{l,m,n,\mathbf{k}} \frac{r_{mn}^{d}}{\omega_{nm}-\omega_{3}}\left[\frac{1}{\omega_{nm}-\omega_{2}}\left(\frac{r_{nl}^{b}r_{lm}^{a}f_{ml}}{\omega_{lm}-\omega_{1}}-\frac{r_{nl}^{a}r_{lm}^{b}f_{ln}}{\omega_{nl}-\omega_{1}}\right)\right]_{;k^{c}} \\ \begin{matrix} & & +i\sum_{l,m,n,\mathbf{k}} \frac{r_{mn}^{d}}{\omega_{nm}-\omega_{3}}\left[\frac{r_{nl}^{c}}{\omega_{lm}-\omega_{2}}\left(\frac{r_{lm}^{a}f_{ml}}{\omega_{lm}-\omega_{1}}\right)_{;k^{b}}-\frac{r_{lm}^{c}}{\omega_{nl}-\omega_{1}}\left(\frac{r_{nl}^{a}f_{ln}}{\omega_{nl}-\omega_{1}}\right)_{;k^{b}}\right] \end{matrix} \\ \begin{matrix} & & -\sum_{m,n,\mathbf{k}} \frac{r_{mn}^{d}}{\omega_{nm}-\omega_{3}}\left[\frac{1}{\omega_{nm}-\omega_{2}}\left(\frac{r_{nm}^{a}f_{mn}}{\omega_{nm}-\omega_{1}}\right)_{;k^{b}}\right] \end{matrix}_{;k^{c}} \end{matrix}$ (S20)

The intraband contribution is described as:

$\begin{matrix} \frac{\chi_{\sigma}^{(3)}}{C}=\frac{1}{i\omega_{3}^{2}}\sum_{l,m,n,\mathbf{k}} \Delta_{nl}^{d}\frac{r_{nl}^{c}}{\omega_{ln}-\omega_{2}}\left(\frac{r_{lm}^{b}r_{mn}^{a}f_{nm}}{\omega_{mn}-\omega_{1}}-\frac{r_{lm}^{a}r_{mn}^{b}f_{ml}}{\omega_{lm}-\omega_{1}}\right)-\frac{1}{\omega_{3}^{2}\omega_{2}}\sum_{m,n,\mathbf{k}} \Delta_{nm}^{d}\left(\frac{r_{nm}^{b}r_{mn}^{a}f_{nm}}{\omega_{mn}-\omega_{1}}\right)_{;k^{c}} \\ \begin{matrix} & & + \end{matrix}\frac{1}{\omega_{3}^{2}}\sum_{m,n,\mathbf{k}} \Delta_{nm}^{d}\frac{r_{nm}^{c}}{\omega_{mn}-\omega_{2}}\left(\frac{r_{mn}^{a}f_{nm}}{\omega_{mn}-\omega_{1}}\right)_{;k^{b}}-\frac{i}{\omega_{3}}\sum_{m,n,l,\mathbf{k}} \frac{(r_{nl}^{c})_{;k^{d}}}{\omega_{mn}-\omega_{2}}\left(\frac{r_{lm}^{b}r_{mn}^{a}f_{nm}}{\omega_{mn}-\omega_{1}}-\frac{r_{lm}^{a}r_{mn}^{b}f_{ml}}{\omega_{lm}-\omega_{1}}\right) \\ \begin{matrix} & & +\frac{1}{\omega_{3}}\sum_{m,n,\mathbf{k}} \frac{(r_{nm}^{c})_{;k^{d}}}{\omega_{mn}-\omega_{2}}\left(\frac{r_{mn}^{a}f_{nm}}{\omega_{mn}-\omega_{1}}\right)_{;k^{b}}- \end{matrix}\frac{1}{i\omega_{3}\omega_{2}}\sum_{m,n,\mathbf{k}} \frac{r_{nm}^{b}r_{mn}^{a}f_{nm}}{\omega_{mn}-\omega_{1}}\left(\frac{\partial\varepsilon_{nm}^{c}}{\partial k^{d}}-\frac{\partial\varepsilon_{nm}^{d}}{\partial k^{c}}\right) \end{matrix}$ (S21)


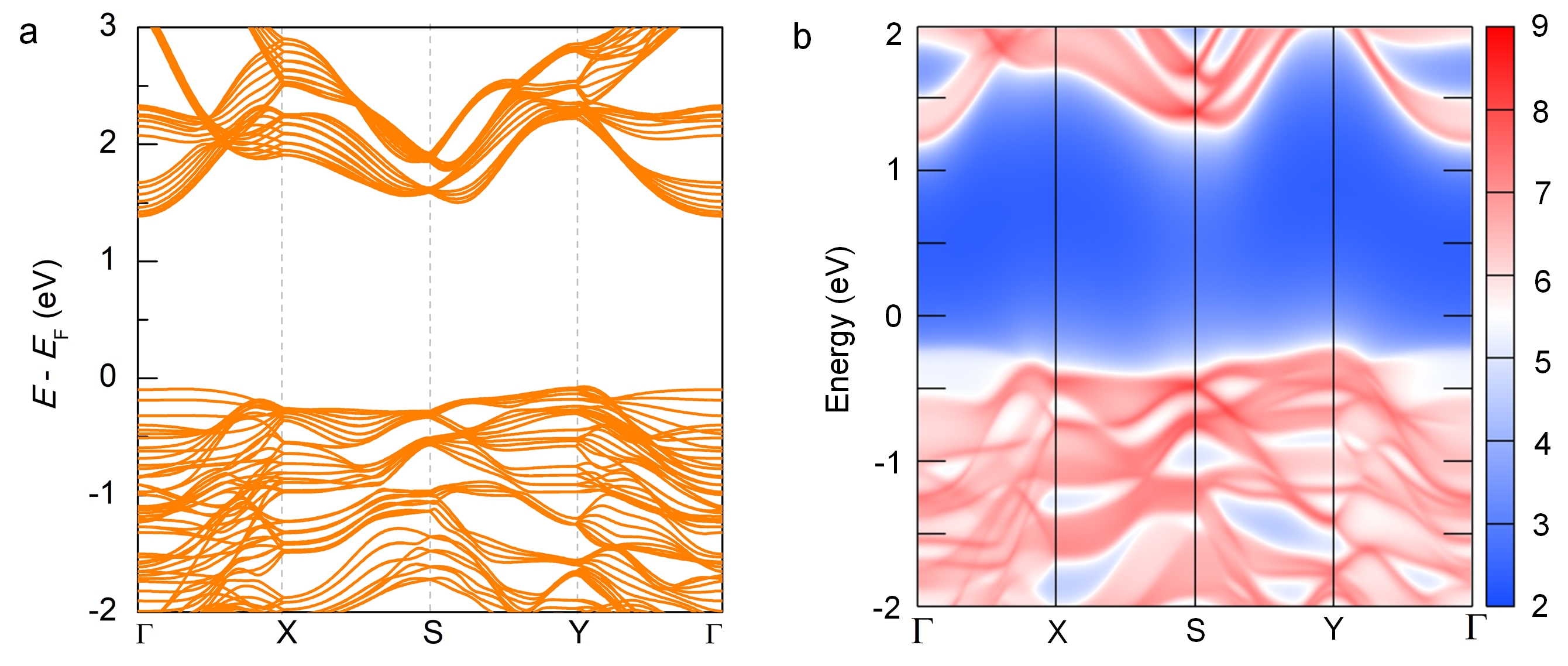


**Figure S17**. The surface states diagram. (a) Band structure of 8 L. (b) The distribution of density of states to study the surface states. To reveal surface state contribution in SHG of layered PdPSe, the surface state is calculated, as shown in **Figure S17**. In comparison with the 8 L band structure, no extra surface states can be observed, which attributes to expansion of orbitals confined in a thin layer without any dangling bonds to form new states on the surface.


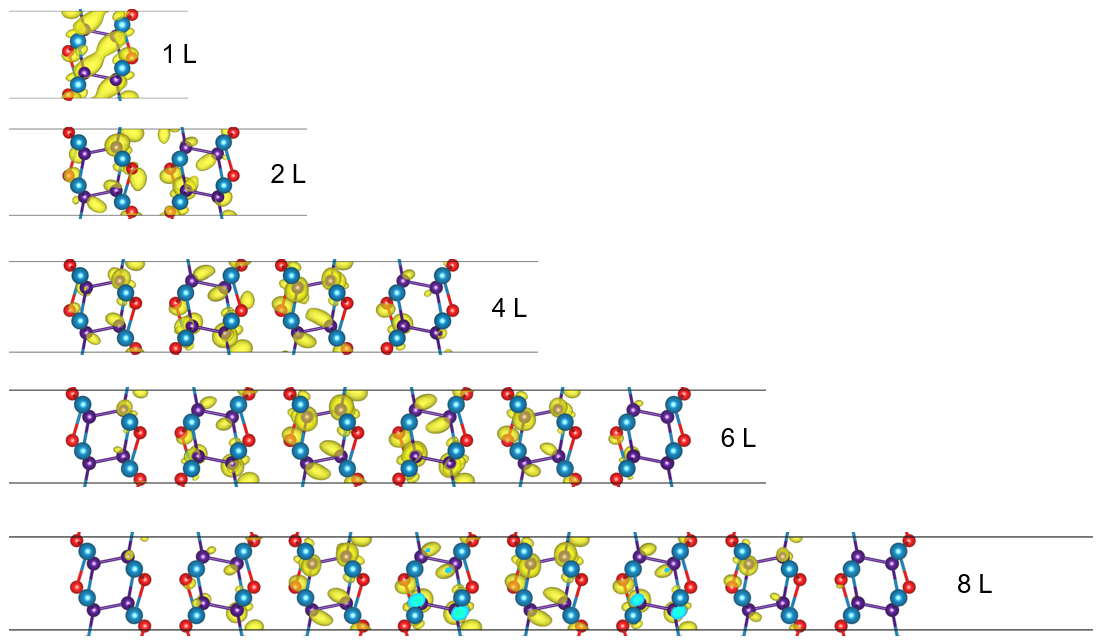


**Figure S18**. The distribution of wavefunctions contributing to SHG in 2 L, 4 L, 6 L and 8 L PdPSe. From the distribution of wavefunctions contributing to SHG, the orbitals tend to be at central layers with the increase of the thickness, which also illustrates that such surface state is not responsible for SHG, as shown in **Figure S18**.


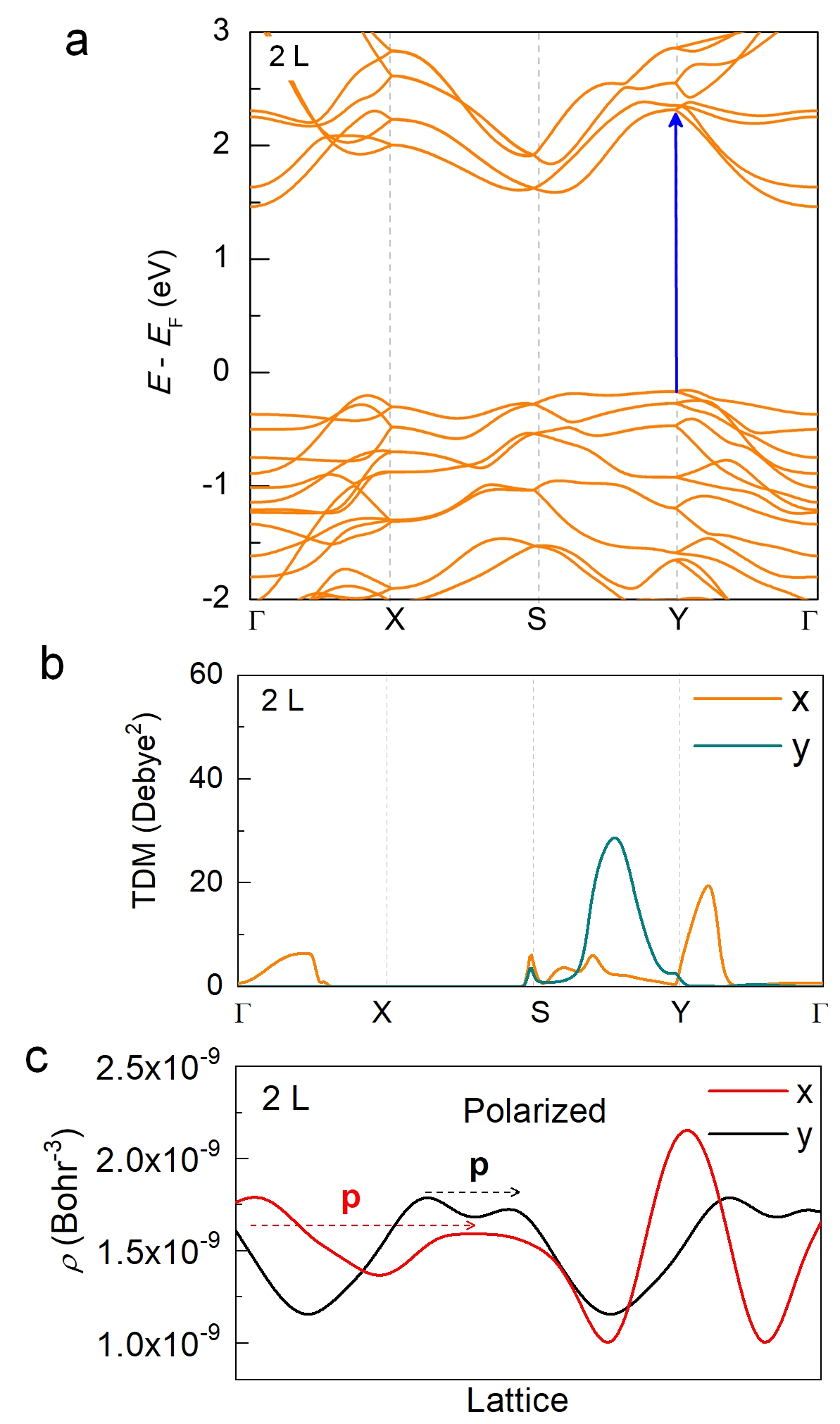


**Figure S19**. (a) The Band structure of 2 L PdPSe. The blue arrow indicates the optical transition including two-photon absorption. (b) Transition dipole moment (TDM) in *x*- and *y*-directions. (c) The polarizations of electronic wavefunction in *x*- and *y*-directions. Arrows indicate the polarization direction. To reveal the physical interpretation for the large anisotropic ratio of nonlinear optics in PdPSe, we further performed some analyses for the optical transition probability and the polarized strength of electronic states which contribute to the SHG signal (2 L as an example), as shown in **Figure S19**. Based on our simulations, the optical transition mainly happens around Y-point in Brillouin zoon (**Figure S19**a). The derived TDM is shown in **Figure S19**b. It is observed that the strength of TDM in the *x*-direction is larger than that in the *y*-direction, which contributes to the large SHG anisotropic ratio. For the underlying physics, the polarization of wavefunctions is studied in **Figure S19**c. From the distribution of the planar-average charge density which contributes to nonlinear optics, we observe evident polarizations in *x-* and *y-* directions. The polarized strength in the *x-* direction is obviously higher than that in the *y*-direction. Such centrosymmetric breaking of the electronic state is the origin for the strong SHG anisotropy. With the increase of the excitation wavelength, the optical transition will happen within S-Y region (**Figure S19**b). Thus, the SHG anisotropic ratio inversion takes place in line with our experimental results (**Figures 4**a-f).


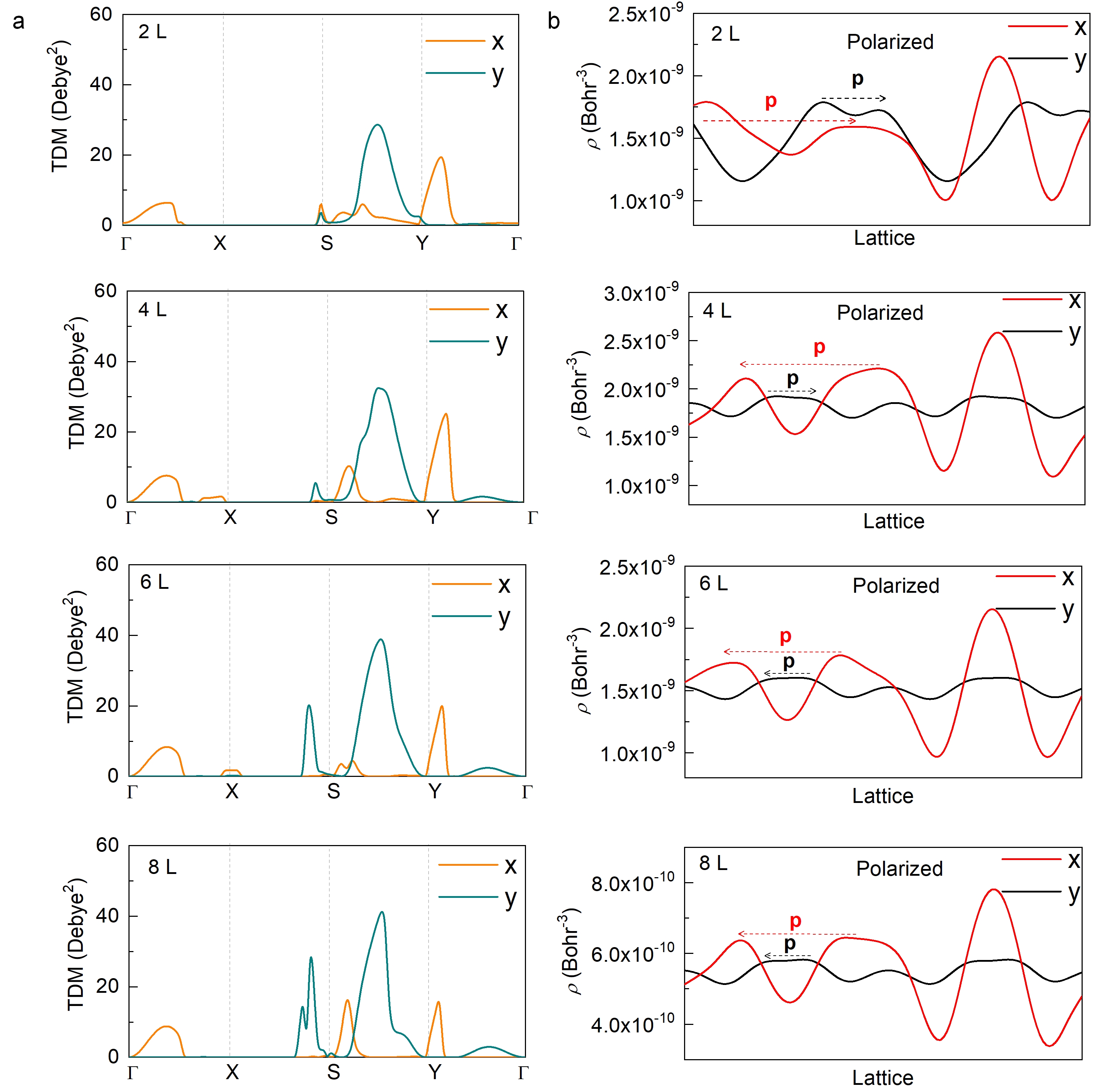


**Figure S20**. Transition dipole moment (a) and planar-average charge density in *x*- and *y*-direction (b) for 2 L, 4 L, 6 L and 8 L PdPSe. **Figure S20** shows the layer-dependent TDM and electronic polarization in 2 L, 4 L, 6 L and 8 L PdPSe. Similarly, the strength of TDM in the *x*-direction around Y-point, is 1.62 Debye^2^ for 2 L, 4 L, 6 L and 8 L PdPSe, while the strengths of TDM are 0.34 Debye2 for 2 L, 0.21 Debye2 for 4 L, 0.13 Debye2 for 6 L and 0.06 Debye2 for 8 L in the *y*-direction, respectively, which evidently demonstrates the layer-dependent anisotropic ratio in PdPSe in line with the experimental results. Further, the polarized charge density (Δρ) also decreases more rapidly from 2 L to 8 L in the *y*- direction than that in the *x*-direction, which is the origin for the layer-dependent nonlinear optical response (**Figures S20**b)


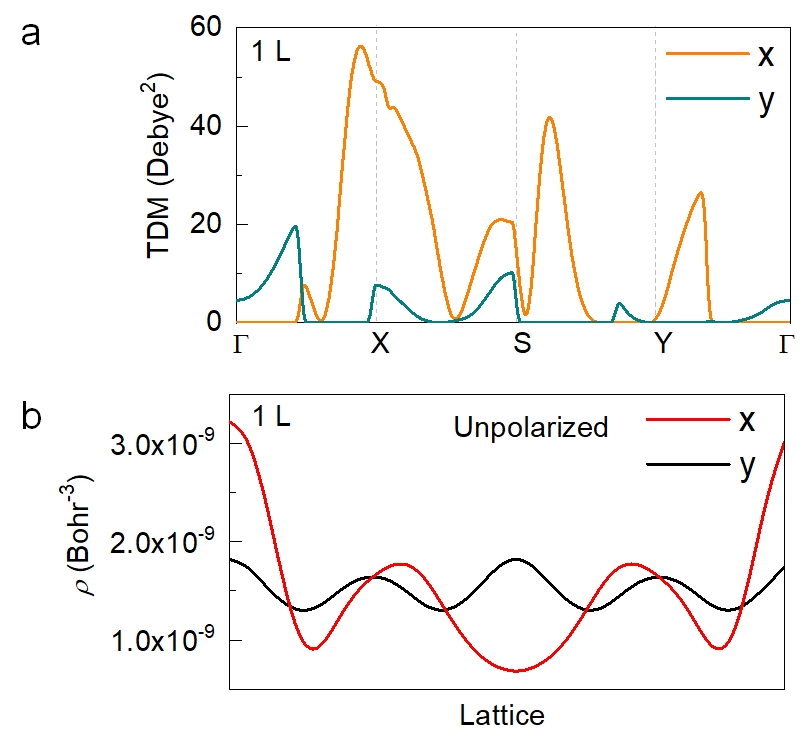


**Figure S21**. (a) TMD and (b) charge density distribution of 1 L PdPSe. The wavefunction of 1 L PdPSe shows a unpolarized character, which is intrinsic reason for the destruction of SHG in 1 L PdPSe, also for all odd layers.


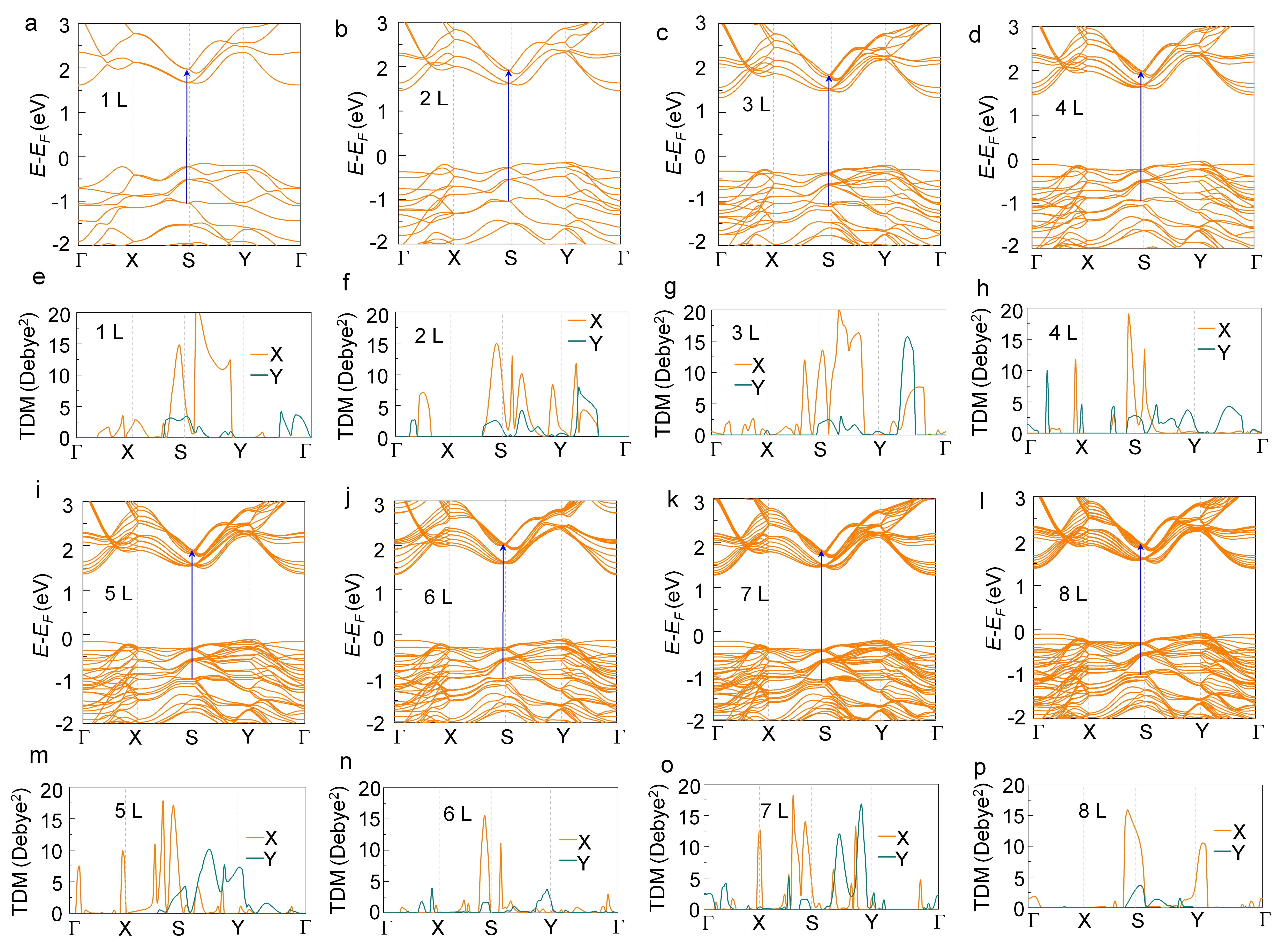


**Figure S22**. Band structures with implication of the three-photon absorption and transition dipole moments (TDMs) in *x*- and *y*-directions for three-photon excitation of 1 L to 8 L PdPSe. The anisotropic ratio in TDMs with the three-photon absorption is 5.43, 6.24, 6.5, 6.73, 7.43, 7.49, 7.5 and 8 for PdPSe with 1 L to 8 L, showing a monotonic increase feature, which is responsible for the layer-dependent $\rho_{THG}$. As shown in **Figure S9**, there is an absorption peak around 490 nm which is responsible for the nonlinear response of SHG (around 980 nm) and THG (around 1400). According to the DFT calculations, we confirm that the SHG process at 980 nm is enhanced by two-photon resonance (along *x*-axis) around Y-point at the band edge, which is marked with the blue arrow in the band structure (**Figure S19**a). The THG enhancement is mainly contributed by the three-photon nonlinear process taking place around S-point, which is marked with the blue arrow in the band structure, as shown in **Figures S22**a-d and i-l. According to the analysis of TDM of 1 L to 8 L PdPSe, we confirm that the strong THG anisotropy between *x*- and *y*-directions stems from the three-photon absorption anisotropy, as shown in **Figures S22**e-h and m-p. The anisotropic ratio of TDM between *x*- and *y*-directions around S-point is 5.43 for 1 L, 6.24 for 2 L, 6.5 for 3 L, 6.73 for 4 L, 7.43 for 5 L, 7.49 for 6 L, 7.5 for 7 L and 8 for 8 L PdPSe. Such transition anisotropies are indeed responsible for the monotonic increase in $\rho_{THG}$ with the layer number.

**Note S14. The damage threshold and air-stability**

We performed power dependent SHG in the PdPSe flake. As shown in **Figure S23**a, it can be found that the damage threshold is around 84 GW/cm^2^, which is much larger than that of the commercial PPLN crystal^45^. **Figure S23**b shows the SHG intensities of pristine PdPSe and PdPSe after 7 days. There is no change for the SHG intensity under the same excitation power, demonstrating its good air-stability.


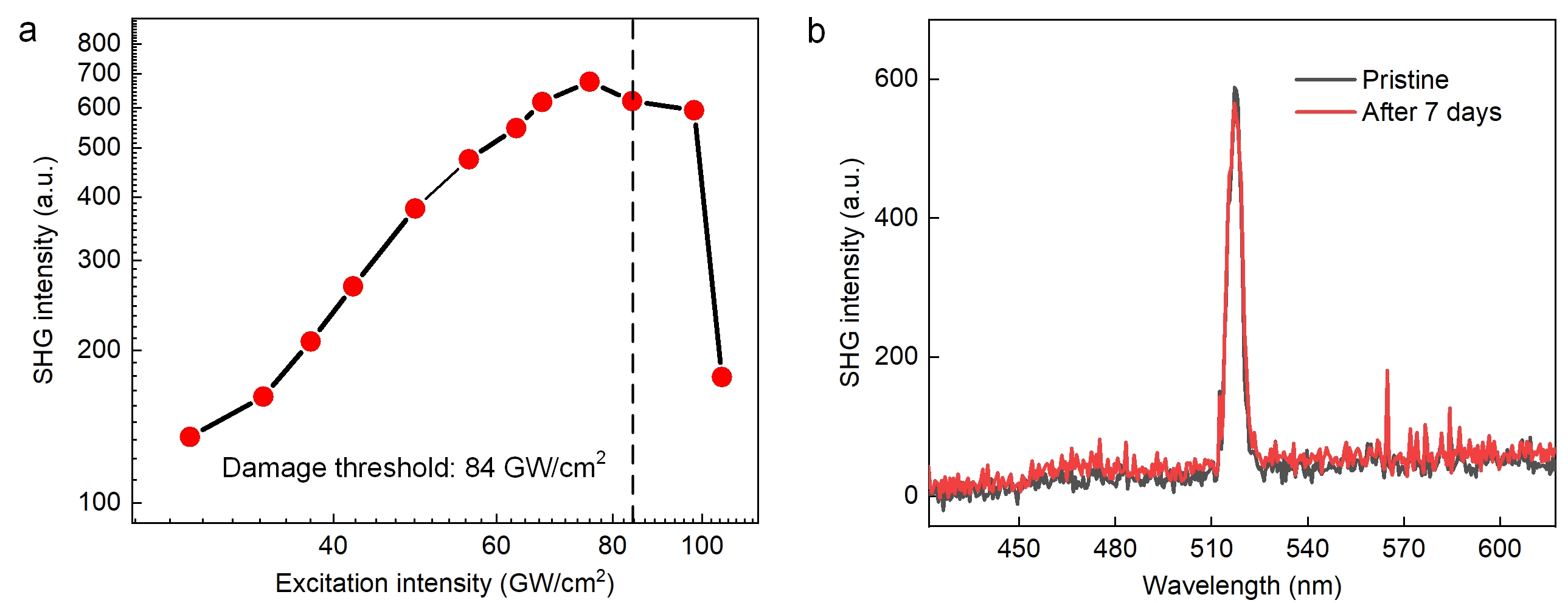


**Figure S23.** (a) The SHG intensity versus the excitation intensity. The damage threshold is around 84 GW/cm^2^. (b) SHG intensities of pristine PdPSe and PdPSe after 7 days under the excitation intensity of 63 GW/cm^2^.

**References**

1. Li, P. *et al.* Penta-PdPSe: A new 2D pentagonal material with highly in-plane optical, electronic, and optoelectronic anisotropy. *Adv. Mater.* **33**, 2102541 (2021).

2. Yang, X. L. & Xie, S. L. Expression of third-order effective nonlinear susceptibility for third-harmonic generation in crystals. *Appl. Opt.* **34**, 6130-6135 (1995).

3. Frisenda, R. *et al.* Micro-reflectance and transmittance spectroscopy: a versatile and powerful tool to characterize 2D materials. *J. Phys. D* **50**, 074002 (2017).

4. Dhakal, K. P. *et al.* Confocal absorption spectral imaging of MoS_2_: optical transitions depending on the atomic thickness of intrinsic and chemically doped MoS_2_. *Nanoscale* **6**, 13028-13035 (2014).

5. Yu, J. *et al.* Giant nonlinear optical activity in two-dimensional palladium diselenide. *Nat. Commun.* **12**, 1083 (2021).

6. Hao, Q. *et al.* Phase identification and strong second harmonic generation in pure ε-InSe and its alloys. *Nano Lett.* **19**, 2634-2640 (2019).

7. Clark, D. J. *et al.* Near bandgap second-order nonlinear optical characteristics of MoS_2_ monolayer transferred on transparent substrates. *Appl. Phys. Lett.* **107**, 131113 (2015).

8. Le, C. T. *et al.* Impact of selenium doping on resonant second-harmonic generation in monolayer MoS_2_. *ACS Photonics* **4**, 38-44 (2017).

9. Susoma, J. *et al.* Second and third harmonic generation in few-layer gallium telluride characterized by multiphoton microscopy. *Appl. Phys. Lett.* **108**, 073103 (2016).

10. Ahmed, S. *et al.* Nonlinear optical activities in two-dimensional gallium sulfide: a comprehensive study. *ACS Nano* **16**, 12390-12402 (2022).

11. Abdelwahab, I. *et al.* Giant second-harmonic generation in ferroelectric NbOI_2_. *Nat. Photon.* **16**, 644-650 (2022).

12. Wang, J.-P. *et al.* Non-centrosymmetric 2D Nb_3_SeI_7_ with high in-plane anisotropy and optical nonlinearity. *Adv. Optical Mater.* **11**, 2300031 (2023).

13. Boyd, R. W. Nonlinear optics. 3rd edn (Academic Press, 2008).

14. Dmitriev, V. G., Gurzadyan, G. G. & Nikogosyan, D. N. Handbook of nonlinear optical crystals (Springer, 1991).

15. Li, D. *et al.* Anisotropic enhancement of second-harmonic generation in monolayer and bilayer MoS_2_ by integrating with TiO_2_ nanowires. *Nano Lett.* **19**, 4195-4204 (2019).

16. Song, Y. *et al.* Extraordinary second harmonic generation in ReS_2_ atomic crystals. *ACS Photonics* **5**, 3485-3491 (2018).

17. Zheng, X. *et al.* Symmetry engineering induced in-plane polarization in MoS_2_ through van der Waals interlayer coupling. *Adv. Funct. Mater.* **32**, 2202658 (2022).

18. Zhu, M. *et al.* Efficient and anisotropic second harmonic generation in few-layer SnS film. *Adv. Opt. Mater.* **9**, 2101200 (2021).

19. Gu, Y. *et al.* Two‐dimensional palladium diselenide with strong in‐plane optical anisotropy and high mobility grown by chemical vapor deposition. *Adv. Mater.* **32**, 1906238 (2020).

20. Cui, Q. *et al.* Strong and anisotropic third-harmonic generation in monolayer and multilayer ReS_2_. *Phys. Rev. B* **95**, 165406 (2017).

21. Autere, A. *et al.* Optical harmonic generation in monolayer group-VI transition metal dichalcogenides. *Phys. Rev. B* **98**, 115426 (2018).

22. Woodward, R. I. *et al.* Characterization of the second- and third-order nonlinear optical susceptibilities of monolayer MoS_2_ using multiphoton microscopy. *2D Materials* **4**, 011006 (2016).

23. Youngblood, N. *et al.* Layer-tunable third-harmonic generation in multilayer black phosphorus. *ACS Photonics* **4**, 8-14 (2017).

24. Popkova, A. A. *et al.* Optical third-harmonic generation in hexagonal boron nitride thin films. *ACS Photonics* **8**, 824-831 (2021).

25. Ning, T. *et al.* Third-harmonic UV generation in silicon nitride nanostructures. *Opt. Express* **21**, 2012-2017 (2013).

26. Gubler, U. & Bosshard, C. Optical third-harmonic generation of fused silica in gas atmosphere: Absolute value of the third-order nonlinear optical susceptibility $\chi^{(3)}$. *Phys. Rev. B* **61**, 10702-10710 (2000).

27. Kulagin, I. A. *et al.* Components of the third-order nonlinear susceptibility tensors in KDP, DKDP and LiNbO_3_ nonlinear optical crystals. *Quantum Electron.* **34**, 657 (2004).

28. Chen, Y. J. & Carter, G. M. Measurement of third order nonlinear susceptibilities by surface plasmons. *Appl. Phys. Lett.* **41**, 307-309 (1982).

29. Sar, H., Gao, J. & Yang, X. In-plane anisotropic third-harmonic generation from germanium arsenide thin flakes. *Sci. Rep.* **10**, 14282 (2020).

30. Dasgupta, A., Gao, J. & Yang, X. Anisotropic third‐harmonic generation in layered germanium selenide. *Laser Photonics Rev.* **14**, 1900416 (2020).

31. Wu, H.-Y., Yen, Y. & Liu, C.-H. Observation of polarization and thickness dependent third-harmonic generation in multilayer black phosphorus. *Appl. Phys. Lett.* **109**, 261902 (2016).

32. Tripathi, R. P. N., Yang, X. & Gao, J. Polarization-dependent optical responses in natural 2D layered mineral teallite. *Sci. Rep.* **11**, 21895 (2021).

33. Sar, H., Gao, J. & Yang, X. 2D layered SiP as anisotropic nonlinear optical material. *Sci. Rep.* **11**, 6372 (2021).

34. Dasgupta, A., Yang, X. & Gao, J. Naturally occurring van der Waals heterostructure lengenbachite with strong in-plane structural and optical anisotropy. *npj 2D Mater. Appl.* **5**, 88 (2021).

35. Dasgupta, A., Gao, J. & Yang, X. Natural van der Waals heterostructure cylindrite with highly anisotropic optical responses. *npj 2D Mater. Appl.* **5**, 74 (2021).

36. Tripathi, R. P. N., Yang, X. & Gao, J. Van der Waals layered mineral getchellite with anisotropic linear and nonlinear optical responses. *Laser Photonics Rev.* **15**, 2100182 (2021).

37. Saynatjoki, A. *et al.* Ultra-strong nonlinear optical processes and trigonal warping in MoS_2_ layers. *Nat. Commun.* **8**, 893 (2017).

38. Malitson, I. H. Interspecimen comparison of the refractive index of fused silica. *J. Opt. Soc. Am.* **55**, 1205-1209 (1965).

39. Karvonen, L. *et al.* Investigation of second-and third-harmonic generation in few-layer gallium selenide by multiphoton microscopy. *Sci. Rep.* **5**, 10334 (2015).

40. Autere, A. *et al.* Optical harmonic generation in monolayer group-VI transition metal dichalcogenides. *Phys. Rev. B* **98**, 115426 (2018).

41. Sun, J., Ruzsinszky, A. & Perdew, J. P. Strongly constrained and appropriately normed semilocal density functional. *Phys. Rev. Lett.* **115**, 036402 (2015).

42. Kresse, G. & Furthmüller, J. Efficiency of ab-initio total energy calculations for metals and semiconductors using a plane-wave basis set. *Comput. Mater. Sci.* **6**, 15-50 (1996).

43. Peng, H. *et al.* Versatile van der Waals density functional based on a meta-generalized gradient approximation. *Phys. Rev. X* **6**, 041005 (2016).

44. Marzari, N. *et al.* Maximally localized Wannier functions: Theory and applications. *Rev. Mod. Phys.* **84**, 1419-1475 (2012).

45. Baudisch, M. *et al.* Performance of MgO:PPLN, KTA, and KNbO_3_ for mid-wave infrared broadband parametric amplification at high average power. *Opt. Lett.* **39**, 5802-5805 (2014).
